# Supplementary material for: A novel comprehensive metric to assess effectiveness of COVID-19 testing: Inter-country comparison and association with geography, government, and policy response
Source: PLoS One. 2021 Mar 5;16(3):e0248176. doi: 10.1371/journal.pone.0248176 (PMC7935311; doi:10.1371/journal.pone.0248176)
Supplement: S1 Table — Raw data inputs, key epidemiological indicators, multipliers, factors, and sub-indices used to compute CovTI on June 3, 2020 among included countries and territories (n = 165). Further details of each variable are described in the text. C = cases, D = total deaths, R = total recovered, A = active cases, P = population (in millions), T = total tests, CFR = case fatality rate, TPR = test positivity rate, mdemsys = multiplier to account for health system capacity and democratic transparency, f1 = factor 1, f2 = factor 2, Inf = true number of infections, Prev = Estimated Period Prevalence = Inf /P, Act = proportion active cases = A/C, DR = detection rate, DRsi = Detection Rate sub-index, TPsi = Test Positivity sub-index, CFsi = Case-Fatality sub-index ACsi = Active Case sub-index, CovTI = COVID-19 Testing Index. OECD = Organization for Economic Development member, BRIC = Brazil, Russia, India, and China. (DOCX) [file pone.0248176.s001.docx]

**S1 Table. Raw data inputs and computed values for the COVID-19 Testing Index (CovTI) on June 3, 2020.** Raw data inputs, key epidemiological indicators, multipliers, factors, and sub-indices used to compute CovTI on June 3, 2020 among eligible countries and territories (n=165). Further details of each variable are described in the text. C=cases, D=total deaths, R=total recovered, A=active cases, P= population (in millions), T=total tests, CFR=case fatality rate, TPR=test positivity rate, I_dem_=Economist Intelligence Unit Democracy Index, I_sys_=Global Health Security Index Detection and Reporting sub-index, m_demsys_=multiplier to account for health system capacity and democratic transparency. Additional columns displayed further down.

| Country or Territory | C | D | R | A | T | P | CFR | TPR | I_dem_ | I_sys_ | m_demsys_ |
| --- | --- | --- | --- | --- | --- | --- | --- | --- | --- | --- | --- |
| Afghanistan | 16,509 | 270 | 1,450 | 14,789 | 40,950 | 38.8 | 3.3% | 40.3% | 2.85 | 44.8 | 3.25 |
| Albania | 1,164 | 33 | 891 | 240 | 15,068 | 2.9 | 3.6% | 7.7% | 5.89 | 74.3 | 2.14 |
| Algeria | 9,626 | 667 | 6,067 | 2,892 |  | 43.8 | 9.9% |  | 4.01 | 12 | 3.35 |
| Argentina | 18,319 | 569 | 5,709 | 12,041 | 164,084 | 45.1 | 6.2% | 11.2% | 7.02 | 74.9 | 1.85 |
| Armenia | 10,009 | 158 | 3,427 | 6,424 | 59,917 | 3.0 | 3.2% | 16.7% | 5.54 | 60.8 | 2.39 |
| Aruba | 101 | 3 | 98 | 0 | 2,134 | 0.1 | 3.0% | 4.7% | 5.4 | 42 | 2.65 |
| Australia | 7,221 | 102 | 6,626 | 493 | 1,494,692 | 25.5 | 1.5% | 0.5% | 9.09 | 97.3 | 1.06 |
| Austria | 16,759 | 669 | 15,629 | 461 | 456,378 | 9.0 | 4.1% | 3.7% | 8.29 | 73.2 | 1.55 |
| Azerbaijan | 5,935 | 71 | 3,564 | 2,300 | 309,901 | 10.1 | 2.0% | 1.9% | 2.75 | 45 | 3.27 |
| Bahamas | 102 | 11 | 49 | 42 | 2,101 | 0.4 | 18.3% | 4.9% | 5.4 | 21.8 | 2.89 |
| Bahrain | 12,311 | 19 | 7,407 | 4,885 | 330,733 | 1.7 | 0.3% | 3.7% | 2.55 | 45.8 | 3.31 |
| Bangladesh | 52,445 | 709 | 11,120 | 40,616 | 333,073 | 164.5 | 2.7% | 15.7% | 5.88 | 50.9 | 2.42 |
| Belarus | 44,255 | 243 | 19,195 | 24,817 | 562,945 | 9.4 | 1.1% | 7.9% | 2.48 | 28.9 | 3.53 |
| Belgium | 58,615 | 9,505 | 15,934 | 33,176 | 884,386 | 11.6 | 32.4% | 6.6% | 7.64 | 62.5 | 1.84 |
| Benin | 244 | 3 | 148 | 93 | 34,682 | 12.1 | 2.0% | 0.7% | 5.09 | 24.2 | 2.94 |
| Bolivia | 10,531 | 343 | 1,137 | 9,051 | 28,239 | 11.7 | 6.5% | 37.3% | 4.84 | 33.1 | 2.89 |
| Bosnia and Herzegovina | 2,535 | 157 | 1,910 | 468 | 66,722 | 3.3 | 7.6% | 3.8% | 4.86 | 41.7 | 2.78 |
| Brazil | 556,668 | 31,278 | 240,627 | 284,763 | 930,013 | 212.4 | 11.2% | 59.9% | 6.86 | 82.4 | 1.80 |
| Brunei | 141 | 2 | 138 | 1 | 19,624 | 0.4 | 1.4% | 0.7% | 5.4 | 30.5 | 2.78 |
| Bulgaria | 2,538 | 144 | 1,123 | 1,271 | 82,914 | 7.0 | 11.3% | 3.1% | 7.03 | 53.3 | 2.10 |
| Burkina Faso | 881 | 53 | 720 | 108 |  | 20.8 | 6.9% |  | 4.04 | 33.3 | 3.09 |
| Cabo Verde | 466 | 5 | 237 | 224 | 1,307 | 0.6 | 2.1% | 35.7% | 5.4 | 9.3 | 3.04 |
| Cambodia | 125 |  | 123 | 2 | 21,087 | 16.7 | 0.0% | 0.6% | 3.53 | 57.7 | 2.93 |
| Cameroon | 6,585 | 200 | 3,676 | 2,709 |  | 26.5 | 5.2% |  | 2.85 | 35.6 | 3.36 |
| Canada | 92,410 | 7,395 | 50,357 | 34,658 | 1,724,250 | 37.7 | 12.8% | 5.4% | 9.22 | 96.4 | 1.04 |
| Central African Republic | 1,069 | 4 | 23 | 1,042 | 15,021 | 4.8 | 0.7% | 7.1% | 1.32 | 17.7 | 3.96 |
| Chad | 803 | 66 | 562 | 175 |  | 16.4 | 10.5% |  | 1.61 | 36.5 | 3.66 |
| Channel Islands | 560 | 46 | 528 | 0 | 10,255 | 0.2 | 8.0% | 5.5% | 5.4 | 42 | 2.65 |
| Chile | 108,686 | 1,188 | 44,946 | 62,552 | 612,772 | 19.1 | 2.2% | 17.7% | 8.08 | 72.7 | 1.61 |
| China | 83,022 | 4,634 | 78,315 | 73 |  | 1,439.3 | 5.6% |  | 2.26 | 48.5 | 3.35 |
| Colombia | 31,833 | 1,009 | 11,142 | 19,682 | 350,213 | 50.8 | 6.3% | 9.1% | 7.13 | 41.7 | 2.22 |
| Comoros | 132 | 2 | 27 | 103 |  | 0.9 | 3.0% |  | 3.15 | 23.2 | 3.43 |
| Congo (Brazzaville) | 611 | 20 | 179 | 412 |  | 5.5 | 6.5% |  | 3.11 | 7 | 3.64 |
| Congo (Democratic Republic) | 3,326 | 72 | 482 | 2,772 |  | 89.2 | 4.3% |  | 1.13 | 25.1 | 3.92 |
| Costa Rica | 1,105 | 10 | 682 | 413 | 27,318 | 5.1 | 1.4% | 4.0% | 8.13 | 56 | 1.80 |
| Côte d'Ivoire | 3,024 | 33 | 1,501 | 1,490 | 28,120 | 26.3 | 2.2% | 10.8% | 6.57 | 72.3 | 1.99 |
| Croatia | 2,246 | 103 | 2,088 | 55 | 67,297 | 4.1 | 4.7% | 3.3% | 2.84 | 10.5 | 3.66 |
| Cuba | 2,092 | 83 | 1,827 | 182 | 108,389 | 11.3 | 4.3% | 1.9% | 4.05 | 44.5 | 2.95 |
| Cyprus | 952 | 17 | 790 | 145 | 117,411 | 1.2 | 2.1% | 0.8% | 7.59 | 44.9 | 2.06 |
| Czech Republic | 9,364 | 323 | 6,686 | 2,355 | 447,957 | 10.7 | 4.6% | 2.1% | 7.69 | 50.7 | 1.97 |
| Denmark | 11,734 | 580 | 10,489 | 665 | 646,086 | 5.8 | 5.2% | 1.8% | 9.22 | 86 | 1.16 |
| Djibouti | 3,779 | 25 | 1,607 | 2,147 | 30,394 | 1.0 | 1.3% | 12.4% | 2.77 | 17 | 3.60 |
| Dominican Republic | 17,752 | 515 | 11,075 | 6,162 | 84,638 | 10.8 | 4.4% | 21.0% | 6.54 | 37.1 | 2.42 |
| Ecuador | 40,414 | 3,438 | 20,019 | 16,957 | 118,754 | 17.6 | 14.7% | 34.0% | 6.33 | 71.2 | 2.06 |
| Egypt | 27,536 | 1,052 | 6,827 | 19,657 | 135,000 | 102.1 | 7.6% | 20.4% | 3.06 | 41.5 | 3.24 |
| El Salvador | 2,653 | 46 | 1,116 | 1,491 | 94,272 | 6.5 | 3.5% | 2.8% | 6.15 | 73.9 | 2.08 |
| Equatorial Guinea | 1,306 | 12 | 200 | 1,094 | 8,268 | 1.4 | 1.8% | 15.8% | 1.92 | 4.4 | 3.97 |
| Estonia | 1,870 | 68 | 1,632 | 170 | 85,175 | 1.3 | 4.0% | 2.2% | 7.9 | 77.6 | 1.59 |
| Eswatini | 294 | 3 | 198 | 93 | 4,994 | 1.2 | 1.5% | 5.9% | 3.14 | 25.5 | 3.41 |
| Ethiopia | 1,344 | 14 | 231 | 1,099 | 116,309 | 114.6 | 2.1% | 1.2% | 3.44 | 33.7 | 3.24 |
| Finland | 6,887 | 320 | 5,500 | 1,067 | 190,800 | 5.5 | 5.5% | 3.6% | 9.25 | 61.6 | 1.45 |
| France | 189,220 | 28,940 | 68,812 | 91,468 | 1,384,633 | 65.3 | 29.6% | 13.7% | 8.12 | 75.3 | 1.57 |
| French Guiana | 517 | 1 | 207 | 309 |  | 0.3 | 0.4% |  | 5.4 | 42 | 2.65 |
| Gabon | 2,803 | 20 | 779 | 2,004 | 14,122 | 2.2 | 1.4% | 19.8% | 3.61 | 6.1 | 3.52 |
| Georgia | 796 | 13 | 634 | 149 | 58,976 | 4.0 | 2.0% | 1.3% | 5.42 | 75 | 2.24 |
| Germany | 184,091 | 8,674 | 166,400 | 9,017 | 3,952,971 | 83.8 | 5.0% | 4.7% | 8.68 | 84.6 | 1.31 |
| Ghana | 8,297 | 38 | 2,986 | 5,273 | 218,425 | 31.0 | 0.9% | 3.8% | 6.63 | 40.5 | 2.36 |
| Greece | 2,937 | 179 | 1,374 | 1,384 | 185,590 | 10.4 | 11.5% | 1.6% | 7.43 | 78.4 | 1.70 |
| Guadeloupe | 162 | 14 | 138 | 10 | 4,137 | 0.4 | 9.2% | 3.9% | 5.4 | 42 | 2.65 |
| Guatemala | 5,336 | 116 | 795 | 4,425 | 31,427 | 17.9 | 4.3% | 17.0% | 5.26 | 50 | 2.59 |
| Guinea | 3,886 | 23 | 2,267 | 1,596 | 14,407 | 13.1 | 1.0% | 27.0% | 3.14 | 57.2 | 3.03 |
| Guinea-Bissau | 1,339 | 8 | 53 | 1,278 | 1,500 | 2.0 | 1.2% | 89.3% | 2.63 | 23.4 | 3.56 |
| Guyana | 153 | 12 | 70 | 71 | 1,658 | 0.8 | 14.6% | 9.2% | 6.15 | 20.3 | 2.72 |
| Haiti | 2,226 | 45 | 24 | 2,157 | 5,270 | 11.4 | 4.0% | 42.2% | 4.57 | 48.3 | 2.78 |
| Honduras | 5,362 | 217 | 549 | 4,596 | 14,790 | 9.9 | 8.1% | 36.3% | 5.42 | 27.7 | 2.81 |
| Hong Kong | 1,094 | 4 | 1,038 | 52 | 202,930 | 7.5 | 0.4% | 0.5% | 6.02 | 78 | 2.06 |
| Hungary | 3,921 | 532 | 2,160 | 1,229 | 189,969 | 9.7 | 19.8% | 2.1% | 6.63 | 55.5 | 2.18 |
| Iceland | 1,806 | 10 | 1,794 | 2 | 61,156 | 0.3 | 0.6% | 3.0% | 9.58 | 37.2 | 1.66 |
| India | 207,191 | 5,829 | 100,285 | 101,077 | 3,966,075 | 1,378.5 | 5.5% | 5.2% | 6.9 | 47.4 | 2.21 |
| Indonesia | 27,549 | 1,663 | 7,935 | 17,951 | 342,464 | 273.2 | 12.1% | 8.0% | 6.48 | 68.1 | 2.06 |
| Iran | 157,562 | 7,942 | 123,077 | 26,543 | 975,936 | 83.9 | 6.1% | 16.1% | 2.38 | 37.7 | 3.45 |
| Iraq | 7,387 | 235 | 3,508 | 3,644 | 238,251 | 40.1 | 6.3% | 3.1% | 3.74 | 42.2 | 3.06 |
| Ireland | 25,066 | 1,658 | 22,089 | 1,319 | 348,416 | 4.9 | 7.0% | 7.2% | 9.24 | 78 | 1.25 |
| Israel | 17,285 | 290 | 14,940 | 2,055 | 582,445 | 8.6 | 1.9% | 3.0% | 7.86 | 52.4 | 1.91 |
| Italy | 233,515 | 33,530 | 160,092 | 39,893 | 3,962,292 | 60.5 | 17.3% | 5.9% | 7.52 | 78.5 | 1.68 |
| Jamaica | 588 | 9 | 322 | 257 | 13,191 | 3.0 | 2.7% | 4.5% | 6.96 | 24.3 | 2.47 |
| Japan | 16,930 | 894 | 14,650 | 1,386 | 296,383 | 126.5 | 5.8% | 5.7% | 7.99 | 70.1 | 1.66 |
| Jordan | 755 | 9 | 549 | 197 | 198,879 | 10.2 | 1.6% | 0.4% | 3.93 | 42.9 | 3.00 |
| Kazakhstan | 11,571 | 44 | 5,941 | 5,586 | 828,377 | 18.8 | 0.7% | 1.4% | 2.94 | 28.2 | 3.43 |
| Kenya | 2,093 | 71 | 499 | 1,523 | 80,054 | 53.6 | 6.8% | 2.6% | 5.18 | 68.6 | 2.38 |
| Kuwait | 28,649 | 226 | 14,281 | 14,142 | 290,013 | 4.3 | 1.6% | 9.9% | 3.93 | 47.5 | 2.95 |
| Kyrgyz Republic | 1,845 | 17 | 1,219 | 609 | 114,353 | 6.5 | 1.4% | 1.6% | 4.89 | 64.7 | 2.50 |
| Latvia | 1,071 | 24 | 760 | 287 | 111,404 | 1.9 | 3.1% | 1.0% | 7.49 | 97.3 | 1.46 |
| Lebanon | 1,242 | 27 | 719 | 496 | 86,244 | 6.8 | 3.6% | 1.4% | 4.36 | 62 | 2.67 |
| Liberia | 311 | 28 | 167 | 116 |  | 5.0 | 14.4% |  | 5.45 | 29.1 | 2.79 |
| Libya | 182 | 5 | 52 | 125 | 6,206 | 6.9 | 5.5% | 2.9% | 2.02 | 36 | 3.56 |
| Lithuania | 1,682 | 71 | 1,249 | 362 | 309,725 | 2.7 | 5.4% | 0.5% | 7.5 | 81.5 | 1.65 |
| Luxembourg | 4,020 | 110 | 3,848 | 62 | 78,026 | 0.6 | 2.8% | 5.2% | 8.81 | 41.7 | 1.80 |
| Madagascar | 845 | 6 | 185 | 654 | 11,954 | 27.6 | 1.4% | 7.1% | 5.64 | 41.9 | 2.59 |
| Malawi | 358 | 4 | 42 | 312 | 5,049 | 19.1 | 2.2% | 7.1% | 5.5 | 23.3 | 2.85 |
| Malaysia | 7,877 | 115 | 6,470 | 1,292 | 560,738 | 32.3 | 1.7% | 1.4% | 7.16 | 73.2 | 1.83 |
| Maldives | 1,841 | 7 | 608 | 1,226 | 11,775 | 0.5 | 0.8% | 15.6% | 5.4 | 25.5 | 2.84 |
| Mali | 1,351 | 78 | 769 | 504 | 3,483 | 20.2 | 9.2% | 38.8% | 4.92 | 25.5 | 2.96 |
| Malta | 620 | 9 | 554 | 57 | 70,693 | 0.4 | 1.6% | 0.9% | 7.95 | 32.9 | 2.12 |
| Martinique | 200 | 14 | 98 | 88 |  | 0.4 | 12.5% |  | 5.4 | 42 | 2.65 |
| Mauritania | 668 | 31 | 55 | 582 | 7,654 | 4.6 | 9.3% | 8.7% | 3.92 | 39.5 | 3.05 |
| Mauritius | 335 | 10 | 322 | 3 | 122,008 | 1.3 | 3.0% | 0.3% | 8.22 | 42.3 | 1.94 |
| Mayotte | 1,986 | 24 | 1,473 | 489 | 7,600 | 0.3 | 1.6% | 26.1% | 5.4 | 42 | 2.65 |
| Mexico | 93,435 | 10,167 | 67,491 | 15,777 | 282,089 | 128.8 | 13.1% | 33.1% | 6.09 | 71.2 | 2.12 |
| Moldova | 8,548 | 307 | 4,738 | 3,503 | 57,003 | 4.0 | 6.1% | 15.0% | 5.75 | 42.9 | 2.55 |
| Mongolia | 185 |  | 44 | 141 | 14,249 | 3.3 | 0.0% | 1.3% | 6.5 | 77.3 | 1.95 |
| Montenegro | 324 | 9 | 315 | 0 | 10,167 | 0.6 | 2.8% | 3.2% | 5.65 | 55.4 | 2.42 |
| Morocco | 7,866 | 206 | 6,410 | 1,250 | 234,651 | 36.9 | 3.1% | 3.4% | 5.1 | 56.8 | 2.54 |
| Mozambique | 307 | 2 | 98 | 207 | 11,239 | 31.1 | 1.3% | 2.7% | 3.65 | 29.3 | 3.24 |
| Myanmar | 232 | 6 | 143 | 83 | 28,304 | 54.4 | 4.0% | 0.8% | 3.55 | 59.2 | 2.90 |
| Nepal | 2,099 | 8 | 266 | 1,825 | 191,688 | 29.1 | 0.8% | 1.1% | 5.28 | 22 | 2.92 |
| Netherlands | 46,647 | 5,967 | N/A | N/A | 359,833 | 17.1 | 25.6% | 13.0% | 9.01 | 86 | 1.22 |
| New Zealand | 1,504 | 22 | 1,481 | 1 | 282,263 | 4.8 | 1.5% | 0.5% | 9.26 | 36.7 | 1.74 |
| Nicaragua | 1,118 | 46 | 370 | 702 |  | 6.6 | 8.2% |  | 3.55 | 39.9 | 3.13 |
| Niger | 960 | 65 | 848 | 47 | 6,068 | 24.1 | 7.1% | 15.8% | 3.29 | 44.4 | 3.14 |
| Nigeria | 10,819 | 314 | 3,240 | 7,265 | 65,885 | 205.5 | 5.8% | 16.4% | 4.12 | 44.6 | 2.93 |
| North Macedonia | 2,391 | 141 | 1,595 | 655 | 31,187 | 2.1 | 8.1% | 7.7% | 5.97 | 41.7 | 2.51 |
| Norway | 8,455 | 237 | 7,727 | 491 | 250,968 | 5.4 | 3.0% | 3.4% | 9.87 | 58.6 | 1.33 |
| Oman | 12,799 | 59 | 2,812 | 9,928 | 100,181 | 5.1 | 0.9% | 12.8% | 3.06 | 41.1 | 3.24 |
| Pakistan | 76,398 | 1,621 | 27,110 | 47,667 | 577,974 | 220.4 | 4.2% | 13.2% | 4.25 | 41.7 | 2.94 |
| Palestine | 451 | 3 | 372 | 76 | 44,876 | 5.1 | 0.8% | 1.0% | 3.89 | 42 | 3.02 |
| Panama | 13,837 | 344 | 9,514 | 3,979 | 67,730 | 4.3 | 3.5% | 20.4% | 7.05 | 44.6 | 2.20 |
| Paraguay | 1,013 | 11 | 498 | 504 | 32,106 | 7.1 | 2.2% | 3.2% | 6.24 | 34.6 | 2.52 |
| Peru | 170,039 | 4,634 | 68,507 | 96,898 | 1,076,659 | 32.9 | 5.5% | 15.8% | 6.6 | 38.3 | 2.39 |
| Philippines | 18,997 | 966 | 4,063 | 13,968 | 366,269 | 109.4 | 10.2% | 5.2% | 6.64 | 63.6 | 2.08 |
| Poland | 24,395 | 1,092 | 11,726 | 11,577 | 945,955 | 37.9 | 8.5% | 2.6% | 6.62 | 61.7 | 2.10 |
| Portugal | 32,895 | 1,436 | 19,869 | 11,590 | 847,181 | 10.2 | 6.7% | 3.9% | 8.03 | 50.5 | 1.89 |
| Qatar | 60,259 | 43 | 36,036 | 24,180 | 231,098 | 2.9 | 0.1% | 26.1% | 3.19 | 32.7 | 3.31 |
| Réunion | 477 | 1 | 411 | 65 | 17,200 | 0.9 | 0.2% | 2.8% | 5.4 | 42 | 2.65 |
| Romania | 19,517 | 1,288 | 13,526 | 4,703 | 448,813 | 19.3 | 8.7% | 4.3% | 6.49 | 42.8 | 2.36 |
| Russia | 423,741 | 5,037 | 186,985 | 231,719 | 11,151,622 | 145.9 | 2.4% | 3.8% | 3.11 | 34.1 | 3.31 |
| Rwanda | 384 | 2 | 269 | 113 | 70,108 | 12.9 | 0.7% | 0.5% | 3.16 | 36 | 3.28 |
| Sao Tome and Principe | 484 | 12 | 68 | 404 | 175 | 0.2 | 5.0% | 100.0% | 5.4 | 42 | 2.65 |
| Saudi Arabia | 89,011 | 549 | 65,790 | 22,672 | 853,987 | 34.8 | 0.8% | 10.4% | 1.93 | 74.4 | 3.12 |
| Senegal | 3,836 | 43 | 1,954 | 1,839 | 44,949 | 16.7 | 2.2% | 8.5% | 5.81 | 35.1 | 2.63 |
| Serbia | 11,454 | 245 | 6,766 | 4,443 | 252,132 | 8.7 | 3.5% | 4.5% | 6.41 | 46.2 | 2.34 |
| Sierra Leone | 896 | 46 | 480 | 370 |  | 8.0 | 8.7% |  | 4.86 | 45.8 | 2.74 |
| Singapore | 35,836 | 24 | 23,175 | 12,637 | 334,691 | 5.8 | 0.1% | 10.7% | 6.02 | 64.5 | 2.22 |
| Slovakia | 1,522 | 28 | 1,372 | 122 | 179,293 | 5.5 | 2.0% | 0.8% | 7.17 | 46 | 2.16 |
| Slovenia | 1,475 | 109 | 1,358 | 8 | 81,066 | 2.1 | 7.4% | 1.8% | 7.5 | 73.7 | 1.74 |
| Somalia | 2,089 | 79 | 361 | 1,649 |  | 15.8 | 7.6% |  | 5.4 | 21.5 | 2.89 |
| South Africa | 35,812 | 755 | 18,313 | 16,744 | 761,534 | 59.2 | 4.0% | 4.7% | 7.24 | 81.5 | 1.71 |
| South Korea | 11,541 | 272 | 10,446 | 823 | 939,851 | 51.3 | 2.5% | 1.2% | 8 | 92.1 | 1.39 |
| South Sudan | 994 | 10 | 6 | 978 | 3,356 | 11.2 | 2.0% | 29.6% | 5.4 | 15.9 | 2.96 |
| Spain | 287,012 | 27,127 | N/A | N/A | 4,063,843 | 46.8 | 18.9% | 7.1% | 8.29 | 83 | 1.43 |
| Sri Lanka | 1,683 | 11 | 823 | 849 | 66,421 | 21.4 | 1.3% | 2.5% | 6.27 | 43 | 2.42 |
| Sudan | 5,310 | 307 | 1,625 | 3,378 | 401 | 43.7 | 11.6% | 100.0% | 2.7 | 7 | 3.74 |
| Sweden | 38,589 | 4,468 | N/A | N/A | 275,500 | 10.1 | 23.2% | 14.0% | 9.39 | 86 | 1.12 |
| Switzerland | 30,874 | 1,920 | 28,500 | 454 | 400,793 | 8.6 | 6.3% | 7.7% | 9.03 | 59.1 | 1.53 |
| Syria | 123 | 6 | 50 | 67 |  | 17.4 | 9.8% |  | 1.43 | 2.7 | 4.11 |
| Taiwan | 443 | 7 | 427 | 9 | 72,683 | 23.8 | 1.6% | 0.6% | 7.73 | 81 | 1.60 |
| Tajikistan | 4,100 | 47 | 2,217 | 1,836 |  | 9.5 | 2.1% |  | 1.93 | 24.1 | 3.73 |
| Tanzania | 509 | 21 | 183 | 305 |  | 59.5 | 8.3% |  | 5.16 | 42 | 2.71 |
| Thailand | 3,083 | 58 | 2,966 | 59 | 420,529 | 69.8 | 1.9% | 0.7% | 6.32 | 81 | 1.95 |
| Togo | 445 | 13 | 230 | 202 | 20,948 | 8.3 | 5.3% | 2.1% | 3.3 | 46.8 | 3.11 |
| Trinidad and Tobago | 117 | 8 | 108 | 1 | 3,195 | 1.4 | 6.9% | 3.7% | 7.16 | 14.7 | 2.53 |
| Tunisia | 1,086 | 48 | 965 | 73 | 52,874 | 11.8 | 4.7% | 2.1% | 6.72 | 26.3 | 2.50 |
| Turkey | 165,555 | 4,585 | 129,921 | 31,049 | 2,103,044 | 84.2 | 3.4% | 7.9% | 4.09 | 45.6 | 2.93 |
| Uganda | 489 |  | 82 | 407 | 96,825 | 45.6 | 0.0% | 0.5% | 5.02 | 50.3 | 2.64 |
| Ukraine | 24,340 | 727 | 10,078 | 13,535 | 371,668 | 43.8 | 6.0% | 6.5% | 5.9 | 36.5 | 2.59 |
| United Arab Emirates | 35,788 | 269 | 18,726 | 16,793 | 2,145,493 | 9.9 | 1.4% | 1.7% | 2.76 | 31.6 | 3.43 |
| United Kingdom | 277,985 | 39,369 | N/A | N/A | 4,615,146 | 67.8 | 28.3% | 6.0% | 8.52 | 87.3 | 1.32 |
| United States | 1,881,205 | 108,059 | 645,974 | 1,127,172 | 18,603,174 | 330.8 | 11.5% | 10.1% | 7.96 | 98.2 | 1.33 |
| Uruguay | 826 | 23 | 691 | 112 | 45,226 | 3.5 | 3.2% | 1.8% | 8.38 | 33.5 | 2.00 |
| Uzbekistan | 3,760 | 15 | 2,908 | 837 | 460,000 | 33.4 | 0.5% | 0.8% | 2.01 | 19.4 | 3.76 |
| Venezuela | 1,819 | 18 | 334 | 1,467 | 987,902 | 28.4 | 2.0% | 0.2% | 2.88 | 8.7 | 3.68 |
| Vietnam | 328 |  | 298 | 30 | 275,000 | 97.2 | 0.0% | 0.1% | 3.08 | 57.4 | 3.04 |
| Yemen | 399 | 87 | 15 | 297 | 120 | 29.7 | 43.6% | 100.0% | 1.95 | 9 | 3.90 |
| Zambia | 1,089 | 7 | 912 | 170 | 28,236 | 18.3 | 0.8% | 3.9% | 5.09 | 21.9 | 2.96 |
| Zimbabwe | 206 | 4 | 29 | 173 | 46,613 | 14.8 | 3.9% | 0.4% | 3.16 | 65.6 | 2.92 |

**S1 Table. Raw data inputs and computed values for the COVID-19 Testing Index (CovTI) on June 3, 2020.** Raw data inputs, key epidemiological indicators, multipliers, factors, and sub-indices used to compute CovTI on June 3, 2020 among eligible countries and territories (n=165). f_1_=factor 1, f_2_=factor 2, Inf= true number of infections, Prev=Estimated Period Prevalence = Inf /P, Act=proportion active cases=A/C, DR=detection rate, DRsi= Detection Rate sub-index, TPsi=Test Positivity sub-index, CFsi= Case-Fatality sub-index ACsi= Active Case sub-index, CovTI=COVID-19 Testing Index. Details of each variable are further described in the text.

| Country or Territory | f_1_ | f_2_ | Inf | Prev | Act | DR | DRsi | TPsi | CFsi | ACsi | CovTI |
| --- | --- | --- | --- | --- | --- | --- | --- | --- | --- | --- | --- |
| Afghanistan | 5.32 | 8.06 | 133,112 | 0.343% | 89.6% | 12.4% | 46.2 | 1.8 | 72.1 | 10.4 | 35.3 |
| Albania | 6.06 | 1.54 | 7,048 | 0.245% | 20.6% | 16.5% | 56.2 | 46.2 | 70.0 | 79.4 | 61.6 |
| Algeria | 23.24 | 5.00 | 223,678 | 0.511% | 30.0% | 4.3% | 19.4 | 20.0 | 37.1 | 70.0 | 33.2 |
| Argentina | 5.73 | 2.23 | 105,049 | 0.233% | 65.7% | 17.4% | 58.2 | 32.7 | 53.7 | 34.3 | 47.4 |
| Armenia | 3.77 | 3.34 | 37,689 | 1.272% | 64.2% | 26.6% | 73.5 | 18.8 | 72.9 | 35.8 | 54.9 |
| Aruba | 7.86 | 1.00 | 794 | 0.744% | 0.0% | 12.7% | 47.1 | 62.3 | 74.3 | 100.0 | 66.1 |
| Australia | 1.50 | 1.00 | 10,811 | 0.042% | 6.8% | 66.8% | 96.5 | 95.3 | 85.9 | 93.2 | 93.5 |
| Austria | 6.18 | 1.00 | 103,635 | 1.151% | 2.8% | 16.2% | 55.5 | 69.3 | 66.3 | 97.2 | 68.7 |
| Azerbaijan | 3.91 | 1.00 | 23,235 | 0.229% | 38.8% | 25.5% | 72.1 | 82.6 | 82.3 | 61.2 | 74.1 |
| Bahamas | 31.15 | 1.00 | 3,177 | 0.809% | 41.2% | 3.2% | 14.8 | 61.5 | 16.0 | 58.8 | 33.2 |
| Bahrain | 3.31 | 1.00 | 40,785 | 2.408% | 39.7% | 30.2% | 77.9 | 68.9 | 97.5 | 60.3 | 76.5 |
| Bangladesh | 3.27 | 3.15 | 171,521 | 0.104% | 77.4% | 30.6% | 78.3 | 20.7 | 76.3 | 22.6 | 55.2 |
| Belarus | 3.53 | 1.57 | 156,362 | 1.655% | 56.1% | 28.3% | 75.7 | 45.6 | 89.6 | 43.9 | 66.1 |
| Belgium | 29.84 | 1.33 | 1,748,920 | 15.098% | 56.6% | 3.4% | 15.4 | 51.5 | 3.9 | 43.4 | 25.9 |
| Benin | 3.61 | 1.00 | 881 | 0.007% | 38.1% | 27.7% | 75.0 | 93.2 | 82.0 | 61.9 | 77.4 |
| Bolivia | 9.42 | 7.46 | 99,223 | 0.851% | 85.9% | 10.6% | 41.2 | 2.4 | 52.1 | 14.1 | 30.2 |
| Bosnia and Herzegovina | 17.25 | 1.00 | 43,718 | 1.332% | 18.5% | 5.8% | 25.2 | 68.4 | 46.8 | 81.5 | 49.4 |
| Brazil | 10.09 | 11.97 | 6,663,977 | 3.138% | 51.2% | 8.4% | 34.1 | 0.3 | 32.5 | 48.8 | 30.0 |
| Brunei | 3.95 | 1.00 | 557 | 0.127% | 0.7% | 25.3% | 71.8 | 93.1 | 86.7 | 99.3 | 84.5 |
| Bulgaria | 11.93 | 1.00 | 30,282 | 0.435% | 50.1% | 8.4% | 34.2 | 73.6 | 32.2 | 49.9 | 44.8 |
| Burkina Faso | 18.59 | 5.00 | 16,379 | 0.079% | 12.3% | 5.4% | 23.6 | 20.0 | 50.4 | 87.7 | 41.1 |
| Cabo Verde | 3.26 | 7.13 | 3,323 | 0.598% | 48.1% | 14.0% | 50.4 | 2.8 | 81.3 | 51.9 | 47.4 |
| Cambodia | 2.93 | 1.00 | 366 | 0.002% | 1.6% | 34.2% | 81.9 | 94.2 | 100.0 | 98.4 | 91.3 |
| Cameroon | 10.21 | 5.00 | 67,206 | 0.254% | 41.1% | 9.8% | 38.7 | 20.0 | 59.7 | 58.9 | 43.2 |
| Canada | 8.31 | 1.07 | 767,749 | 2.036% | 37.5% | 12.0% | 45.2 | 58.5 | 27.8 | 62.5 | 47.8 |
| Central African Republic | 3.96 | 1.42 | 4,231 | 0.088% | 97.5% | 25.3% | 71.7 | 49.1 | 92.8 | 2.5 | 57.6 |
| Chad | 30.08 | 5.00 | 24,153 | 0.148% | 21.8% | 3.3% | 15.3 | 20.0 | 35.0 | 78.2 | 32.8 |
| Channel Islands | 21.74 | 1.09 | 12,172 | 7.008% | 0.0% | 4.6% | 20.5 | 57.9 | 44.9 | 100.0 | 48.8 |
| Chile | 1.76 | 3.55 | 385,548 | 2.019% | 57.6% | 28.2% | 75.6 | 17.0 | 80.4 | 42.4 | 58.2 |
| China | 18.72 | 5.00 | 1,553,780 | 0.108% | 0.1% | 5.3% | 23.4 | 20.0 | 57.2 | 99.9 | 44.8 |
| Colombia | 7.03 | 1.82 | 223,705 | 0.440% | 61.8% | 14.2% | 50.9 | 40.3 | 53.1 | 38.2 | 46.7 |
| Comoros | 5.20 | 5.00 | 687 | 0.079% | 78.0% | 19.2% | 61.7 | 20.0 | 73.9 | 22.0 | 47.9 |
| Congo (Brazzaville) | 11.91 | 5.00 | 7,277 | 0.132% | 67.4% | 8.4% | 34.3 | 20.0 | 52.0 | 32.6 | 34.6 |
| Congo (Democratic Republic) | 8.48 | 5.00 | 28,197 | 0.032% | 83.3% | 11.8% | 44.6 | 20.0 | 64.9 | 16.7 | 38.1 |
| Costa Rica | 1.80 | 1.00 | 1,984 | 0.039% | 37.4% | 55.7% | 93.8 | 66.7 | 86.5 | 62.6 | 80.7 |
| Croatia | 3.22 | 2.15 | 9,747 | 0.037% | 49.3% | 31.0% | 78.8 | 34.1 | 80.6 | 50.7 | 64.6 |
| Cuba | 9.13 | 1.00 | 20,496 | 0.499% | 2.4% | 11.0% | 42.2 | 71.6 | 62.5 | 97.6 | 63.2 |
| Côte d'Ivoire | 14.54 | 1.00 | 30,411 | 0.268% | 8.7% | 6.9% | 29.1 | 82.4 | 64.8 | 91.3 | 59.3 |
| Cyprus | 3.69 | 1.00 | 3,508 | 0.291% | 15.2% | 27.1% | 74.3 | 92.2 | 81.0 | 84.8 | 81.3 |
| Czech Republic | 6.79 | 1.00 | 63,602 | 0.594% | 25.1% | 14.7% | 52.1 | 81.1 | 63.1 | 74.9 | 64.7 |
| Denmark | 5.75 | 1.00 | 67,454 | 1.165% | 5.7% | 17.4% | 58.1 | 83.4 | 59.2 | 94.3 | 70.6 |
| Djibouti | 3.60 | 2.49 | 13,618 | 1.381% | 56.8% | 27.8% | 75.0 | 28.8 | 87.6 | 43.2 | 61.9 |
| Dominican Republic | 7.02 | 4.19 | 124,620 | 1.150% | 34.7% | 14.2% | 50.9 | 12.3 | 64.1 | 65.3 | 48.7 |
| Ecuador | 17.55 | 6.81 | 709,294 | 4.027% | 42.0% | 5.7% | 24.8 | 3.3 | 23.1 | 58.0 | 26.8 |
| Egypt | 12.37 | 4.08 | 340,532 | 0.334% | 71.4% | 8.1% | 33.3 | 13.0 | 46.6 | 28.6 | 30.9 |
| El Salvador | 3.60 | 1.00 | 9,548 | 0.147% | 56.2% | 27.8% | 75.1 | 75.5 | 70.7 | 43.8 | 68.0 |
| Equatorial Guinea | 3.97 | 3.16 | 5,181 | 0.371% | 83.8% | 25.2% | 71.6 | 20.6 | 83.2 | 16.2 | 52.7 |
| Estonia | 5.80 | 1.00 | 10,838 | 0.817% | 9.1% | 17.3% | 57.8 | 80.3 | 67.0 | 90.9 | 70.8 |
| Eswatini | 3.48 | 1.18 | 1,023 | 0.088% | 31.6% | 28.7% | 76.2 | 55.5 | 86.1 | 68.4 | 72.5 |
| Ethiopia | 3.37 | 1.00 | 4,530 | 0.004% | 81.8% | 29.7% | 77.3 | 89.1 | 81.2 | 18.2 | 68.6 |
| Finland | 6.73 | 1.00 | 46,346 | 0.837% | 15.5% | 14.9% | 52.4 | 69.7 | 57.7 | 84.5 | 63.4 |
| France | 23.96 | 2.73 | 4,533,162 | 6.947% | 48.3% | 4.2% | 18.8 | 25.5 | 5.2 | 51.7 | 24.0 |
| French Guiana | 2.65 | 5.00 | 2,585 | 0.868% | 59.8% | 20.0% | 63.2 | 20.0 | 96.2 | 40.2 | 56.6 |
| Gabon | 3.52 | 3.97 | 11,127 | 0.501% | 71.5% | 25.2% | 71.6 | 13.7 | 86.7 | 28.5 | 54.4 |
| Georgia | 3.67 | 1.00 | 2,919 | 0.073% | 18.7% | 27.3% | 74.4 | 87.4 | 81.8 | 81.3 | 79.9 |
| Germany | 6.20 | 1.00 | 1,140,458 | 1.362% | 4.9% | 16.1% | 55.4 | 62.8 | 60.9 | 95.1 | 65.9 |
| Ghana | 2.36 | 1.00 | 19,552 | 0.063% | 63.6% | 42.4% | 88.0 | 68.4 | 91.2 | 36.4 | 74.4 |
| Greece | 10.37 | 1.00 | 30,460 | 0.292% | 47.1% | 9.6% | 38.3 | 85.4 | 31.6 | 52.9 | 49.3 |
| Guadeloupe | 22.87 | 1.00 | 3,704 | 0.926% | 6.2% | 4.4% | 19.6 | 67.6 | 39.8 | 93.8 | 48.1 |
| Guatemala | 5.62 | 3.40 | 29,986 | 0.168% | 82.9% | 17.8% | 58.9 | 18.3 | 64.7 | 17.1 | 43.6 |
| Guinea | 3.03 | 5.39 | 20,963 | 0.160% | 41.1% | 18.5% | 60.4 | 6.7 | 90.4 | 58.9 | 55.4 |
| Guinea-Bissau | 3.56 | 17.85 | 23,906 | 1.218% | 95.4% | 5.6% | 24.4 | 0.0 | 88.7 | 4.6 | 28.4 |
| Guyana | 21.32 | 1.85 | 3,263 | 0.415% | 46.4% | 4.7% | 20.9 | 39.7 | 23.1 | 53.6 | 31.7 |
| Haiti | 5.62 | 8.45 | 18,805 | 0.165% | 96.9% | 11.8% | 44.7 | 1.5 | 66.7 | 3.1 | 32.1 |
| Honduras | 11.38 | 7.25 | 61,033 | 0.617% | 85.7% | 8.8% | 35.5 | 2.7 | 44.5 | 14.3 | 26.5 |
| Hong Kong | 2.06 | 1.00 | 2,253 | 0.030% | 4.8% | 48.6% | 91.2 | 94.8 | 96.2 | 95.2 | 93.7 |
| Hungary | 29.53 | 1.00 | 115,790 | 1.198% | 31.3% | 3.4% | 15.6 | 81.4 | 13.9 | 68.7 | 39.0 |
| Iceland | 1.66 | 1.00 | 2,995 | 0.878% | 0.1% | 60.3% | 95.1 | 74.4 | 94.6 | 99.9 | 91.8 |
| India | 6.21 | 1.04 | 1,285,994 | 0.093% | 48.8% | 16.1% | 55.3 | 59.3 | 57.7 | 51.2 | 55.8 |
| Indonesia | 12.45 | 1.61 | 343,044 | 0.126% | 65.2% | 8.0% | 33.1 | 44.7 | 29.9 | 34.8 | 35.1 |
| Iran | 17.40 | 3.23 | 2,742,055 | 3.269% | 16.8% | 5.7% | 25.0 | 19.9 | 54.5 | 83.2 | 41.5 |
| Iraq | 9.73 | 1.00 | 71,877 | 0.179% | 49.3% | 10.3% | 40.2 | 73.3 | 53.4 | 50.7 | 51.5 |
| Ireland | 8.29 | 1.44 | 207,913 | 4.216% | 5.3% | 12.1% | 45.3 | 48.7 | 49.7 | 94.7 | 56.7 |
| Israel | 3.20 | 1.00 | 55,280 | 0.640% | 11.9% | 31.3% | 79.1 | 74.3 | 82.7 | 88.1 | 80.6 |
| Italy | 24.09 | 1.18 | 5,626,334 | 9.304% | 17.1% | 4.2% | 18.7 | 55.5 | 17.7 | 82.9 | 38.7 |
| Jamaica | 3.78 | 1.00 | 2,222 | 0.075% | 43.7% | 26.5% | 73.4 | 64.0 | 76.2 | 56.3 | 68.7 |
| Japan | 8.77 | 1.14 | 148,520 | 0.117% | 8.2% | 11.4% | 43.4 | 56.5 | 56.3 | 91.8 | 58.3 |
| Jordan | 3.58 | 1.00 | 2,702 | 0.027% | 26.1% | 27.9% | 75.3 | 96.3 | 85.1 | 73.9 | 81.2 |
| Kazakhstan | 3.43 | 1.00 | 39,649 | 0.211% | 48.3% | 29.2% | 76.8 | 87.0 | 92.9 | 51.7 | 77.0 |
| Kenya | 8.08 | 1.00 | 16,911 | 0.032% | 72.8% | 12.4% | 46.1 | 77.0 | 50.7 | 27.2 | 49.5 |
| Kuwait | 2.95 | 1.98 | 84,443 | 1.981% | 49.4% | 33.9% | 81.7 | 37.2 | 85.6 | 50.6 | 67.4 |
| Kyrgyz Republic | 2.50 | 1.00 | 4,615 | 0.071% | 33.0% | 40.0% | 86.5 | 85.1 | 87.1 | 67.0 | 82.4 |
| Latvia | 3.27 | 1.00 | 3,504 | 0.186% | 26.8% | 30.6% | 78.3 | 90.8 | 73.6 | 73.2 | 78.9 |
| Lebanon | 5.80 | 1.00 | 7,198 | 0.105% | 39.9% | 17.3% | 57.8 | 86.6 | 69.6 | 60.1 | 66.4 |
| Liberia | 25.10 | 5.00 | 7,807 | 0.155% | 37.3% | 4.0% | 18.1 | 20.0 | 23.8 | 62.7 | 28.5 |
| Libya | 9.79 | 1.00 | 1,782 | 0.026% | 68.7% | 10.2% | 40.0 | 74.6 | 57.7 | 31.3 | 48.7 |
| Lithuania | 6.95 | 1.00 | 11,694 | 0.429% | 21.5% | 14.4% | 51.3 | 94.7 | 58.4 | 78.5 | 66.8 |
| Luxembourg | 4.92 | 1.03 | 19,768 | 3.164% | 1.5% | 20.3% | 63.8 | 59.7 | 75.7 | 98.5 | 72.3 |
| Madagascar | 2.59 | 1.41 | 2,186 | 0.008% | 77.4% | 38.7% | 85.5 | 49.3 | 86.8 | 22.6 | 65.9 |
| Malawi | 3.18 | 1.42 | 1,138 | 0.006% | 87.2% | 31.5% | 79.3 | 49.2 | 80.0 | 12.8 | 60.1 |
| Malaysia | 2.67 | 1.00 | 21,063 | 0.065% | 16.4% | 37.4% | 84.6 | 86.9 | 84.0 | 83.6 | 84.7 |
| Maldives | 2.84 | 3.13 | 5,757 | 1.067% | 66.6% | 32.0% | 79.8 | 20.9 | 92.7 | 33.4 | 61.3 |
| Mali | 17.11 | 7.76 | 23,119 | 0.115% | 37.3% | 5.8% | 25.3 | 2.1 | 39.8 | 62.7 | 31.1 |
| Malta | 3.07 | 1.00 | 1,906 | 0.432% | 9.2% | 32.5% | 80.3 | 91.6 | 85.2 | 90.8 | 85.7 |
| Martinique | 18.52 | 5.00 | 3,704 | 0.987% | 44.0% | 5.4% | 23.7 | 20.0 | 28.7 | 56.0 | 30.4 |
| Mauritania | 14.14 | 1.75 | 9,443 | 0.204% | 87.1% | 7.1% | 29.8 | 41.8 | 39.5 | 12.9 | 30.8 |
| Mauritius | 5.78 | 1.00 | 1,937 | 0.152% | 0.9% | 17.3% | 57.9 | 97.3 | 74.0 | 99.1 | 77.2 |
| Mayotte | 3.20 | 5.23 | 10,379 | 3.816% | 24.6% | 19.1% | 61.6 | 7.3 | 85.2 | 75.4 | 58.2 |
| Mexico | 23.10 | 6.62 | 2,158,556 | 1.676% | 16.9% | 4.3% | 19.5 | 3.6 | 27.0 | 83.1 | 30.5 |
| Moldova | 9.15 | 3.00 | 78,214 | 1.938% | 41.0% | 10.9% | 42.1 | 22.3 | 54.4 | 59.0 | 44.0 |
| Mongolia | 1.95 | 1.00 | 360 | 0.011% | 76.2% | 51.4% | 92.3 | 87.8 | 100.0 | 23.8 | 79.3 |
| Montenegro | 6.73 | 1.00 | 2,180 | 0.347% | 0.0% | 14.9% | 52.4 | 72.7 | 75.7 | 100.0 | 70.7 |
| Morocco | 6.66 | 1.00 | 52,394 | 0.142% | 15.9% | 15.0% | 52.8 | 71.5 | 73.2 | 84.1 | 66.9 |
| Mozambique | 3.24 | 1.00 | 993 | 0.003% | 67.4% | 30.9% | 78.7 | 76.1 | 87.8 | 32.6 | 70.8 |
| Myanmar | 7.51 | 1.00 | 1,741 | 0.003% | 35.8% | 13.3% | 48.6 | 92.1 | 66.9 | 64.2 | 64.1 |
| Nepal | 2.92 | 1.00 | 6,121 | 0.021% | 86.9% | 34.3% | 82.0 | 89.6 | 92.7 | 13.1 | 71.9 |
| Netherlands | 15.55 | 2.59 | 725,289 | 4.234% |  | 6.4% | 27.5 | 27.4 | 7.7 | 50.0 | 28.0 |
| New Zealand | 2.55 | 1.00 | 3,838 | 0.080% | 0.1% | 39.2% | 85.9 | 94.8 | 86.4 | 99.9 | 90.6 |
| Nicaragua | 12.89 | 5.00 | 14,415 | 0.218% | 62.8% | 7.8% | 32.1 | 20.0 | 43.9 | 37.2 | 33.1 |
| Niger | 21.29 | 3.16 | 20,441 | 0.085% | 4.9% | 4.7% | 20.9 | 20.6 | 49.1 | 95.1 | 41.3 |
| Nigeria | 8.52 | 3.28 | 92,153 | 0.045% | 67.2% | 11.7% | 44.4 | 19.4 | 56.0 | 32.8 | 39.4 |
| North Macedonia | 14.78 | 1.53 | 35,350 | 1.697% | 27.4% | 6.8% | 28.7 | 46.5 | 44.4 | 72.6 | 44.2 |
| Norway | 3.73 | 1.00 | 31,504 | 0.582% | 5.8% | 26.8% | 73.9 | 71.4 | 74.3 | 94.2 | 77.5 |
| Oman | 3.24 | 2.56 | 41,492 | 0.815% | 77.6% | 30.8% | 78.6 | 27.9 | 91.2 | 22.4 | 59.7 |
| Pakistan | 6.23 | 2.64 | 476,104 | 0.216% | 62.4% | 16.0% | 55.2 | 26.7 | 65.4 | 37.6 | 48.0 |
| Palestine | 3.02 | 1.00 | 1,364 | 0.027% | 16.9% | 33.1% | 80.9 | 90.4 | 92.3 | 83.1 | 85.5 |
| Panama | 5.48 | 4.09 | 75,759 | 1.759% | 28.8% | 18.3% | 59.9 | 13.0 | 70.5 | 71.2 | 54.9 |
| Paraguay | 2.74 | 1.00 | 2,777 | 0.039% | 49.8% | 36.5% | 83.9 | 72.9 | 80.6 | 50.2 | 74.3 |
| Peru | 6.51 | 3.16 | 1,107,711 | 3.365% | 57.0% | 15.4% | 53.6 | 20.6 | 58.0 | 43.0 | 45.8 |
| Philippines | 10.56 | 1.04 | 200,619 | 0.183% | 73.5% | 9.5% | 37.7 | 59.5 | 36.2 | 26.5 | 39.5 |
| Poland | 9.42 | 1.00 | 229,822 | 0.607% | 47.5% | 10.6% | 41.2 | 77.3 | 42.7 | 52.5 | 51.0 |
| Portugal | 8.24 | 1.00 | 270,901 | 2.656% | 35.2% | 12.1% | 45.5 | 67.8 | 51.0 | 64.8 | 54.9 |
| Qatar | 3.31 | 5.22 | 314,252 | 10.929% | 40.1% | 19.2% | 61.7 | 7.4 | 98.8 | 59.9 | 57.9 |
| Réunion | 2.65 | 1.00 | 1,262 | 0.141% | 13.6% | 37.8% | 84.9 | 75.8 | 97.6 | 86.4 | 85.9 |
| Romania | 15.60 | 1.00 | 304,470 | 1.582% | 24.1% | 6.4% | 27.4 | 64.7 | 41.9 | 75.9 | 47.5 |
| Russia | 3.94 | 1.00 | 1,668,909 | 1.144% | 54.7% | 25.4% | 71.9 | 68.4 | 78.8 | 45.3 | 67.3 |
| Rwanda | 3.28 | 1.00 | 1,259 | 0.010% | 29.4% | 30.5% | 78.2 | 94.7 | 92.9 | 70.6 | 82.9 |
| Sao Tome and Principe | 6.56 | 20.00 | 9,680 | 4.427% | 83.5% | 5.0% | 22.1 | 0.0 | 60.9 | 16.5 | 24.3 |
| Saudi Arabia | 3.12 | 2.08 | 278,133 | 0.800% | 25.5% | 32.0% | 79.8 | 35.3 | 92.1 | 74.5 | 72.3 |
| Senegal | 2.94 | 1.71 | 11,293 | 0.068% | 47.9% | 34.0% | 81.7 | 42.6 | 80.6 | 52.1 | 67.7 |
| Serbia | 5.01 | 1.00 | 57,406 | 0.657% | 38.8% | 20.0% | 63.1 | 63.5 | 70.5 | 61.2 | 64.3 |
| Sierra Leone | 14.04 | 5.00 | 12,583 | 0.158% | 41.3% | 7.1% | 30.0 | 20.0 | 41.7 | 58.7 | 36.1 |
| Singapore | 2.22 | 2.14 | 79,592 | 1.362% | 35.3% | 45.0% | 89.5 | 34.3 | 99.0 | 64.7 | 75.4 |
| Slovakia | 3.97 | 1.00 | 6,035 | 0.111% | 8.0% | 25.2% | 71.7 | 91.9 | 81.9 | 92.0 | 81.8 |
| Slovenia | 12.86 | 1.00 | 18,973 | 0.913% | 0.5% | 7.8% | 32.2 | 83.4 | 47.6 | 99.5 | 59.0 |
| Somalia | 10.94 | 5.00 | 22,847 | 0.144% | 78.9% | 9.1% | 36.7 | 20.0 | 46.9 | 21.1 | 32.3 |
| South Africa | 3.61 | 1.00 | 129,256 | 0.218% | 46.8% | 27.7% | 75.0 | 62.5 | 67.3 | 53.2 | 66.6 |
| South Korea | 3.29 | 1.00 | 37,939 | 0.074% | 7.1% | 30.4% | 78.2 | 88.4 | 77.6 | 92.9 | 83.0 |
| South Sudan | 2.98 | 5.92 | 5,888 | 0.053% | 98.4% | 16.9% | 57.0 | 5.2 | 81.8 | 1.6 | 40.5 |
| Spain | 13.53 | 1.41 | 3,883,230 | 8.306% |  | 7.4% | 30.9 | 49.3 | 15.1 | 50.0 | 35.2 |
| Sri Lanka | 2.42 | 1.00 | 4,067 | 0.019% | 50.4% | 41.4% | 87.4 | 77.6 | 87.7 | 49.6 | 77.9 |
| Sudan | 21.63 | 20.00 | 114,849 | 0.263% | 63.6% | 4.6% | 20.6 | 0.0 | 31.5 | 36.4 | 21.8 |
| Sweden | 12.97 | 2.80 | 500,639 | 4.961% |  | 7.7% | 32.0 | 24.6 | 9.9 | 50.0 | 29.7 |
| Switzerland | 9.54 | 1.54 | 294,394 | 3.404% | 1.5% | 10.5% | 40.8 | 46.3 | 53.2 | 98.5 | 55.9 |
| Syria | 20.05 | 5.00 | 2,466 | 0.014% | 54.5% | 5.0% | 22.1 | 20.0 | 37.7 | 45.5 | 29.5 |
| Taiwan | 2.52 | 1.00 | 1,117 | 0.005% | 2.0% | 39.7% | 86.2 | 94.1 | 85.1 | 98.0 | 89.9 |
| Tajikistan | 4.27 | 5.00 | 20,500 | 0.216% | 44.8% | 20.0% | 63.2 | 20.0 | 81.3 | 55.2 | 56.6 |
| Tanzania | 11.16 | 5.00 | 5,683 | 0.010% | 59.9% | 9.0% | 36.1 | 20.0 | 43.8 | 40.1 | 35.2 |
| Thailand | 3.66 | 1.00 | 11,298 | 0.016% | 1.9% | 27.3% | 74.4 | 92.9 | 82.5 | 98.1 | 84.5 |
| Togo | 9.10 | 1.00 | 4,047 | 0.049% | 45.4% | 11.0% | 42.3 | 80.9 | 58.6 | 54.6 | 55.7 |
| Trinidad and Tobago | 17.32 | 1.00 | 2,027 | 0.145% | 0.9% | 5.8% | 25.1 | 69.3 | 50.2 | 99.1 | 53.8 |
| Tunisia | 11.07 | 1.00 | 12,021 | 0.102% | 6.7% | 9.0% | 36.3 | 81.4 | 62.3 | 93.3 | 61.9 |
| Turkey | 8.12 | 1.57 | 1,343,543 | 1.595% | 18.8% | 12.3% | 46.0 | 45.5 | 71.1 | 81.2 | 58.0 |
| Uganda | 2.64 | 1.00 | 1,292 | 0.003% | 83.2% | 37.8% | 84.9 | 95.1 | 100.0 | 16.8 | 76.3 |
| Ukraine | 7.73 | 1.31 | 188,075 | 0.430% | 55.6% | 12.9% | 47.6 | 52.0 | 55.0 | 44.4 | 49.3 |
| United Arab Emirates | 3.43 | 1.00 | 122,781 | 1.243% | 46.9% | 29.1% | 76.7 | 84.6 | 86.8 | 53.1 | 75.6 |
| United Kingdom | 18.73 | 1.20 | 5,206,157 | 7.673% |  | 5.3% | 23.4 | 54.8 | 5.9 | 50.0 | 31.5 |
| United States | 7.65 | 2.02 | 14,389,136 | 4.350% | 59.9% | 13.1% | 48.0 | 36.4 | 31.7 | 40.1 | 40.8 |
| Uruguay | 5.58 | 1.00 | 4,607 | 0.133% | 13.6% | 17.9% | 59.2 | 83.3 | 72.5 | 86.4 | 72.1 |
| Uzbekistan | 3.76 | 1.00 | 14,155 | 0.042% | 22.3% | 26.6% | 73.5 | 92.2 | 95.0 | 77.7 | 82.4 |
| Venezuela | 3.68 | 1.00 | 6,686 | 0.024% | 80.6% | 27.2% | 74.3 | 98.2 | 82.0 | 19.4 | 69.7 |
| Vietnam | 3.04 | 1.00 | 998 | 0.001% | 9.1% | 32.9% | 80.7 | 98.8 | 100.0 | 90.9 | 90.2 |
| Yemen | 85.14 | 20.00 | 33,969 | 0.114% | 74.4% | 1.2% | 5.7 | 0.0 | 1.3 | 25.6 | 7.7 |
| Zambia | 2.96 | 1.00 | 3,229 | 0.018% | 15.6% | 33.7% | 81.5 | 68.0 | 92.7 | 84.4 | 81.6 |
| Zimbabwe | 5.68 | 1.00 | 1,169 | 0.008% | 84.0% | 17.6% | 58.6 | 95.7 | 67.8 | 16.0 | 59.3 |

**S1 Table. Raw data inputs and computed values for the COVID-19 Testing Index (CovTI) on June 3, 2020.** Raw data inputs, key epidemiological indicators, multipliers, factors, and sub-indices used to compute CovTI on June 3, 2020 among eligible countries and territories (n=165). OECD= Organization for Economic Development member, BRIC= Brazil, Russia, India, and China.

| Country or Territory | Global Rank | OECD or BRIC | Island | Type of government |
| --- | --- | --- | --- | --- |
| Afghanistan | 156 |  |  | unitary |
| Albania | 87 |  |  | unitary |
| Algeria | 162 |  |  | unitary |
| Argentina | 134 |  |  | federation |
| Armenia | 111 |  |  | unitary |
| Aruba | 75 |  | yes | unitary |
| Australia | 3 | OECD | yes | federation |
| Austria | 63 | OECD |  | federation |
| Azerbaijan | 50 |  |  | unitary |
| Bahamas | 161 |  | yes | unitary |
| Bahrain | 44 |  | limited land | unitary |
| Bangladesh | 108 |  |  | unitary |
| Belarus | 76 |  |  | unitary |
| Belgium | 184 | OECD |  | federation |
| Benin | 40 |  |  | unitary |
| Bolivia | 175 |  |  | unitary |
| Bosnia and Herzegovina | 123 |  |  | federation |
| Brazil | 176 | BRIC |  | federation |
| Brunei | 21 |  | co-island | unitary |
| Bulgaria | 138 |  |  | unitary |
| Burkina Faso | 147 |  |  | unitary |
| Cabo Verde | 135 |  | yes | unitary |
| Cambodia | 7 |  |  | unitary |
| Cameroon | 143 |  |  | unitary |
| Canada | 132 | OECD |  | federation |
| Central African Republic | 100 |  |  | unitary |
| Chad | 164 |  |  | unitary |
| Channel Islands | 126 |  | yes | unitary |
| Chile | 97 | OECD |  | unitary |
| China | 139 | BRIC |  | unitary |
| Colombia | 136 | OECD |  | unitary |
| Comoros | 131 |  | yes | federation |
| Congo (Brazzaville) | 160 |  |  | unitary |
| Congo (Democratic Republic) | 154 |  |  | unitary |
| Costa Rica | 32 |  |  | unitary |
| Croatia | 80 |  |  | unitary |
| Cuba | 84 |  |  | unitary |
| Côte d'Ivoire | 92 |  | yes | unitary |
| Cyprus | 30 |  | yes | unitary |
| Czech Republic | 79 | OECD |  | unitary |
| Denmark | 59 | OECD |  | unitary |
| Djibouti | 85 |  |  | unitary |
| Dominican Republic | 128 |  | co-island | unitary |
| Ecuador | 182 |  |  | unitary |
| Egypt | 170 |  |  | unitary |
| El Salvador | 67 |  |  | unitary |
| Equatorial Guinea | 116 |  |  | unitary |
| Estonia | 57 | OECD |  | unitary |
| Eswatini | 51 |  |  | unitary |
| Ethiopia | 66 |  |  | federation |
| Finland | 83 | OECD |  | unitary |
| France | 186 | OECD |  | unitary |
| French Guiana | 103 |  |  | unitary |
| Gabon | 113 |  |  | unitary |
| Georgia | 34 |  |  | unitary |
| Germany | 78 | OECD |  | federation |
| Ghana | 48 |  |  | unitary |
| Greece | 125 | OECD |  | unitary |
| Guadeloupe | 129 |  | yes | unitary |
| Guatemala | 142 |  |  | unitary |
| Guinea | 107 |  |  | unitary |
| Guinea-Bissau | 180 |  |  | unitary |
| Guyana | 167 |  |  | unitary |
| Haiti | 166 |  | co-island | unitary |
| Honduras | 183 |  |  | unitary |
| Hong Kong | 1 |  | limited land | unitary |
| Hungary | 152 | OECD |  | unitary |
| Iceland | 4 | OECD | yes | unitary |
| India | 105 | BRIC |  | federation |
| Indonesia | 159 |  | archipelago | unitary |
| Iran | 145 |  |  | unitary |
| Iraq | 118 |  |  | federation |
| Ireland | 101 | OECD | co-island | unitary |
| Israel | 33 | OECD |  | unitary |
| Italy | 153 | OECD |  | unitary |
| Jamaica | 64 |  | yes | unitary |
| Japan | 95 | OECD | archipelago | unitary |
| Jordan | 31 |  |  | unitary |
| Kazakhstan | 43 |  |  | unitary |
| Kenya | 122 |  |  | unitary |
| Kuwait | 69 |  |  | unitary |
| Kyrgyz Republic | 26 |  |  | unitary |
| Latvia | 36 | OECD |  | unitary |
| Lebanon | 74 |  |  | unitary |
| Liberia | 179 |  |  | unitary |
| Libya | 127 |  |  | unitary |
| Lithuania | 72 | OECD |  | unitary |
| Luxembourg | 52 | OECD |  | unitary |
| Madagascar | 77 |  | yes | unitary |
| Malawi | 90 |  |  | unitary |
| Malaysia | 20 |  |  | federation |
| Maldives | 88 |  | yes | unitary |
| Mali | 169 |  |  | unitary |
| Malta | 18 |  | yes | unitary |
| Martinique | 174 |  | yes | unitary |
| Mauritania | 171 |  |  | unitary |
| Mauritius | 41 |  | yes | unitary |
| Mayotte | 96 |  | yes | unitary |
| Mexico | 173 | OECD |  | federation |
| Moldova | 141 |  |  | unitary |
| Mongolia | 35 |  |  | unitary |
| Montenegro | 58 |  |  | unitary |
| Morocco | 71 |  |  | unitary |
| Mozambique | 56 |  |  | unitary |
| Myanmar | 82 |  |  | unitary |
| Nepal | 55 |  |  | federation |
| Netherlands | 181 | OECD |  | unitary |
| New Zealand | 10 | OECD | yes | unitary |
| Nicaragua | 163 |  |  | unitary |
| Niger | 146 |  |  | unitary |
| Nigeria | 151 |  |  | federation |
| North Macedonia | 140 |  |  | unitary |
| Norway | 39 | OECD |  | unitary |
| Oman | 91 |  |  | unitary |
| Pakistan | 130 |  |  | federation |
| Palestine | 19 |  |  | unitary |
| Panama | 112 |  |  | unitary |
| Paraguay | 49 |  |  | unitary |
| Peru | 137 |  |  | unitary |
| Philippines | 150 |  | archipelago | unitary |
| Poland | 119 | OECD |  | unitary |
| Portugal | 110 | OECD |  | unitary |
| Qatar | 99 |  |  | unitary |
| Réunion | 17 |  | yes | unitary |
| Romania | 133 |  |  | unitary |
| Russia | 70 | BRIC |  | federation |
| Rwanda | 25 |  |  | unitary |
| Sao Tome and Principe | 185 |  | yes | unitary |
| Saudi Arabia | 53 |  |  | unitary |
| Senegal | 68 |  |  | unitary |
| Serbia | 81 |  |  | unitary |
| Sierra Leone | 155 |  |  | unitary |
| Singapore | 47 |  | limited land | unitary |
| Slovakia | 28 | OECD |  | unitary |
| Slovenia | 94 | OECD |  | unitary |
| Somalia | 165 |  |  | federation |
| South Africa | 73 |  |  | unitary |
| South Korea | 24 | OECD |  | unitary |
| South Sudan | 149 |  |  | federation |
| Spain | 157 | OECD |  | unitary |
| Sri Lanka | 38 |  | yes | unitary |
| Sudan | 187 |  |  | federation |
| Sweden | 177 | OECD |  | unitary |
| Switzerland | 104 | OECD |  | federation |
| Syria | 178 |  |  | unitary |
| Taiwan | 12 |  | yes | unitary |
| Tajikistan | 102 |  |  | unitary |
| Tanzania | 158 |  |  | unitary |
| Thailand | 22 |  |  | unitary |
| Togo | 106 |  |  | unitary |
| Trinidad and Tobago | 114 |  | yes | unitary |
| Tunisia | 86 |  |  | unitary |
| Turkey | 98 | OECD |  | unitary |
| Uganda | 45 |  |  | unitary |
| Ukraine | 124 |  |  | unitary |
| United Arab Emirates | 46 |  |  | federation |
| United Kingdom | 168 | OECD |  | unitary |
| United States | 148 | OECD |  | federation |
| Uruguay | 54 |  |  | unitary |
| Uzbekistan | 27 |  |  | unitary |
| Venezuela | 60 |  |  | federation |
| Vietnam | 11 |  |  | unitary |
| Yemen | 188 |  |  | unitary |
| Zambia | 29 |  |  | unitary |
| Zimbabwe | 93 |  |  | unitary |
