# Supplementary material for: A novel comprehensive metric to assess effectiveness of COVID-19 testing: Inter-country comparison and association with geography, government, and policy response
Source: PLoS One. 2021 Mar 5;16(3):e0248176. doi: 10.1371/journal.pone.0248176 (PMC7935311; doi:10.1371/journal.pone.0248176)
Supplement: S2 Table — CovTI as of June 3, 2020; island status; form of government; OECD membership; COVID-19 testing policy as of May 13, 2020; and COVID-19 contact tracing policy as of May 13, 2020 among included countries and territories with complete data (n = 147). Further details of each variable are described in the text. (DOCX) [file pone.0248176.s002.docx]

**S2 Table. Dataset for bivariate and multiple linear regression analyses.** CovTI as of June 3, 2020; island status; form of government; OECD membership; COVID-19 testing policy as of May 13, 2020; and COVID-19 contact tracing policy as of May 13, 2020 among included countries and territories with complete data (n=147). Further details of each variable are described in the text.

| Country or Territory | CovTI | Island status | Form of government | OECD Member | Testing policy | Contact tracing policy |
| --- | --- | --- | --- | --- | --- | --- |
| Afghanistan | 35.3 | non-island | unitary | No | Limited contact tracing | limited testing |
| Albania | 61.6 | non-island | unitary | No | Limited contact tracing | symptomatic testing |
| Algeria | 33.2 | non-island | unitary | No | No contact tracing | No testing policy |
| Argentina | 47.4 | non-island | federation | No | Extensive contact tracing | limited testing |
| Aruba | 66.1 | island-like | unitary | No | Limited contact tracing | limited testing |
| Australia | 93.5 | island-like | federation | Yes | Limited contact tracing | limited testing |
| Austria | 68.7 | non-island | federation | Yes | Extensive contact tracing | symptomatic testing |
| Azerbaijan | 74.1 | non-island | unitary | No | Limited contact tracing | symptomatic testing |
| Bahrain | 76.5 | island-like | unitary | No | Extensive contact tracing | public testing |
| Bangladesh | 55.2 | non-island | unitary | No | Limited contact tracing | symptomatic testing |
| Belarus | 66.1 | non-island | unitary | No | Extensive contact tracing | public testing |
| Belgium | 25.9 | non-island | federation | Yes | Limited contact tracing | symptomatic testing |
| Benin | 77.4 | non-island | unitary | No | Extensive contact tracing | symptomatic testing |
| Bolivia | 30.2 | non-island | unitary | No | Limited contact tracing | No testing policy |
| Bosnia and Herzegovina | 49.4 | non-island | federation | No | No contact tracing | symptomatic testing |
| Brazil | 30.0 | non-island | federation | No | No contact tracing | limited testing |
| Brunei | 84.5 | island-like | unitary | No | Limited contact tracing | symptomatic testing |
| Bulgaria | 44.8 | non-island | unitary | No | Extensive contact tracing | limited testing |
| Burkina Faso | 41.1 | non-island | unitary | No | Extensive contact tracing | limited testing |
| Cabo Verde | 47.4 | island-like | unitary | No | Limited contact tracing | symptomatic testing |
| Cambodia | 91.3 | non-island | unitary | No | Limited contact tracing | symptomatic testing |
| Cameroon | 43.2 | non-island | unitary | No | Limited contact tracing | symptomatic testing |
| Canada | 47.8 | non-island | federation | Yes | Limited contact tracing | public testing |
| Central African Republic | 57.6 | non-island | unitary | No | Limited contact tracing | limited testing |
| Chad | 32.8 | non-island | unitary | No | Limited contact tracing | limited testing |
| Chile | 58.2 | non-island | unitary | Yes | Extensive contact tracing | symptomatic testing |
| China | 44.8 | non-island | unitary | No | Extensive contact tracing | public testing |
| Colombia | 46.7 | non-island | unitary | Yes | Extensive contact tracing | symptomatic testing |
| Congo (Brazzaville) | 34.6 | non-island | unitary | No | Limited contact tracing | limited testing |
| Congo (Democratic Republic) | 38.1 | non-island | unitary | No | Limited contact tracing | limited testing |
| Costa Rica | 80.7 | non-island | unitary | No | Limited contact tracing | limited testing |
| Croatia | 63.2 | non-island | unitary | No | Extensive contact tracing | public testing |
| Cuba | 59.3 | island-like | unitary | No | Extensive contact tracing | limited testing |
| Côte d'Ivoire | 64.6 | non-island | unitary | No | Limited contact tracing | symptomatic testing |
| Cyprus | 81.3 | island-like | unitary | No | Extensive contact tracing | public testing |
| Czech Republic | 64.7 | non-island | unitary | Yes | Extensive contact tracing | symptomatic testing |
| Denmark | 70.6 | non-island | unitary | Yes | Extensive contact tracing | public testing |
| Djibouti | 61.9 | non-island | unitary | No | Extensive contact tracing | public testing |
| Dominican Republic | 48.7 | island-like | unitary | No | Limited contact tracing | symptomatic testing |
| Ecuador | 26.8 | non-island | unitary | No | Limited contact tracing | public testing |
| Egypt | 30.9 | non-island | unitary | No | Extensive contact tracing | limited testing |
| El Salvador | 68.0 | non-island | unitary | No | No contact tracing | public testing |
| Estonia | 70.8 | non-island | unitary | Yes | No contact tracing | symptomatic testing |
| Eswatini | 72.5 | non-island | unitary | No | Limited contact tracing | symptomatic testing |
| Ethiopia | 68.6 | non-island | federation | No | Extensive contact tracing | limited testing |
| Finland | 63.4 | non-island | unitary | Yes | Limited contact tracing | limited testing |
| France | 24.0 | non-island | unitary | Yes | Extensive contact tracing | symptomatic testing |
| Gabon | 54.4 | non-island | unitary | No | Extensive contact tracing | public testing |
| Georgia | 79.9 | non-island | unitary | No | Extensive contact tracing | symptomatic testing |
| Germany | 65.9 | non-island | federation | Yes | Limited contact tracing | public testing |
| Ghana | 74.4 | non-island | unitary | No | Extensive contact tracing | public testing |
| Greece | 49.3 | non-island | unitary | Yes | No contact tracing | symptomatic testing |
| Guatemala | 43.6 | non-island | unitary | No | No contact tracing | symptomatic testing |
| Guinea | 55.4 | non-island | unitary | No | Limited contact tracing | symptomatic testing |
| Guyana | 31.7 | non-island | unitary | No | Extensive contact tracing | limited testing |
| Haiti | 32.1 | island-like | unitary | No | No contact tracing | limited testing |
| Honduras | 26.5 | non-island | unitary | No | Limited contact tracing | limited testing |
| Hong Kong | 93.7 | island-like | unitary | No | Extensive contact tracing | symptomatic testing |
| Hungary | 39.0 | non-island | unitary | Yes | Extensive contact tracing | limited testing |
| Iceland | 91.8 | island-like | unitary | Yes | Extensive contact tracing | public testing |
| India | 55.8 | non-island | federation | No | Extensive contact tracing | public testing |
| Indonesia | 35.1 | island-like | unitary | No | Limited contact tracing | limited testing |
| Iran | 41.5 | non-island | unitary | No | No contact tracing | No testing policy |
| Iraq | 51.5 | non-island | federation | No | No contact tracing | symptomatic testing |
| Ireland | 56.7 | island-like | unitary | Yes | Limited contact tracing | limited testing |
| Israel | 80.6 | non-island | unitary | Yes | Extensive contact tracing | limited testing |
| Italy | 38.7 | non-island | unitary | Yes | Extensive contact tracing | symptomatic testing |
| Jamaica | 68.7 | island-like | unitary | No | Limited contact tracing | limited testing |
| Japan | 58.3 | island-like | unitary | Yes | Limited contact tracing | limited testing |
| Jordan | 81.2 | non-island | unitary | No | Limited contact tracing | symptomatic testing |
| Kazakhstan | 77.0 | non-island | unitary | No | Extensive contact tracing | public testing |
| Kenya | 49.5 | non-island | unitary | No | Limited contact tracing | limited testing |
| Kuwait | 67.4 | non-island | unitary | No | Extensive contact tracing | symptomatic testing |
| Kyrgyz Republic | 82.4 | non-island | unitary | No | Limited contact tracing | limited testing |
| Lebanon | 66.4 | non-island | unitary | No | Limited contact tracing | limited testing |
| Liberia | 28.5 | non-island | unitary | No | Limited contact tracing | limited testing |
| Libya | 48.7 | non-island | unitary | No | Limited contact tracing | symptomatic testing |
| Lithuania | 66.8 | non-island | unitary | Yes | Extensive contact tracing | symptomatic testing |
| Luxembourg | 72.3 | non-island | unitary | Yes | Extensive contact tracing | public testing |
| Madagascar | 65.9 | island-like | unitary | No | Limited contact tracing | limited testing |
| Malawi | 60.1 | non-island | unitary | No | Extensive contact tracing | limited testing |
| Malaysia | 84.7 | non-island | federation | No | Extensive contact tracing | public testing |
| Mali | 31.1 | non-island | unitary | No | Extensive contact tracing | limited testing |
| Mauritania | 30.8 | non-island | unitary | No | Extensive contact tracing | No testing policy |
| Mauritius | 77.2 | island-like | unitary | No | Extensive contact tracing | limited testing |
| Mexico | 30.5 | non-island | federation | Yes | Limited contact tracing | limited testing |
| Moldova | 44.0 | non-island | unitary | No | Extensive contact tracing | symptomatic testing |
| Mongolia | 79.3 | non-island | unitary | No | Extensive contact tracing | limited testing |
| Morocco | 66.9 | non-island | unitary | No | No contact tracing | symptomatic testing |
| Mozambique | 70.8 | non-island | unitary | No | Extensive contact tracing | limited testing |
| Myanmar | 64.1 | non-island | unitary | No | Limited contact tracing | symptomatic testing |
| Nepal | 71.9 | non-island | federation | No | Extensive contact tracing | limited testing |
| Netherlands | 28.0 | non-island | unitary | Yes | Limited contact tracing | limited testing |
| New Zealand | 90.6 | island-like | unitary | Yes | Extensive contact tracing | symptomatic testing |
| Nicaragua | 33.1 | non-island | unitary | No | No contact tracing | limited testing |
| Niger | 41.3 | non-island | unitary | No | Limited contact tracing | limited testing |
| Nigeria | 39.4 | non-island | federation | No | Extensive contact tracing | limited testing |
| Norway | 77.5 | non-island | unitary | Yes | Limited contact tracing | limited testing |
| Oman | 59.7 | non-island | unitary | No | Extensive contact tracing | symptomatic testing |
| Pakistan | 48.0 | non-island | federation | No | Extensive contact tracing | symptomatic testing |
| Palestine | 85.5 | non-island | unitary | No | Limited contact tracing | limited testing |
| Panama | 54.9 | non-island | unitary | No | Limited contact tracing | symptomatic testing |
| Paraguay | 74.3 | non-island | unitary | No | Limited contact tracing | symptomatic testing |
| Peru | 45.8 | non-island | unitary | No | Limited contact tracing | symptomatic testing |
| Philippines | 39.5 | island-like | unitary | No | Extensive contact tracing | symptomatic testing |
| Poland | 51.0 | non-island | unitary | Yes | No contact tracing | limited testing |
| Portugal | 54.9 | non-island | unitary | Yes | Limited contact tracing | public testing |
| Qatar | 57.9 | non-island | unitary | No | Extensive contact tracing | public testing |
| Romania | 47.5 | non-island | unitary | No | Limited contact tracing | symptomatic testing |
| Russia | 67.3 | non-island | federation | No | Extensive contact tracing | public testing |
| Rwanda | 82.9 | non-island | unitary | No | Extensive contact tracing | public testing |
| Saudi Arabia | 72.3 | non-island | unitary | No | Extensive contact tracing | symptomatic testing |
| Senegal | 67.7 | non-island | unitary | No | Extensive contact tracing | symptomatic testing |
| Serbia | 64.3 | non-island | unitary | No | Extensive contact tracing | limited testing |
| Sierra Leone | 36.1 | non-island | unitary | No | Extensive contact tracing | symptomatic testing |
| Singapore | 75.4 | island-like | unitary | No | Extensive contact tracing | symptomatic testing |
| Slovakia | 81.8 | non-island | unitary | Yes | Extensive contact tracing | limited testing |
| Slovenia | 59.0 | non-island | unitary | Yes | Extensive contact tracing | symptomatic testing |
| Somalia | 32.3 | non-island | federation | No | No contact tracing | limited testing |
| South Africa | 66.6 | non-island | unitary | No | Extensive contact tracing | public testing |
| South Korea | 83.0 | non-island | unitary | Yes | Extensive contact tracing | public testing |
| South Sudan | 40.5 | non-island | federation | No | Extensive contact tracing | symptomatic testing |
| Spain | 35.2 | non-island | unitary | Yes | Limited contact tracing | symptomatic testing |
| Sri Lanka | 77.9 | island-like | unitary | No | Extensive contact tracing | symptomatic testing |
| Sudan | 21.8 | non-island | federation | No | Limited contact tracing | symptomatic testing |
| Sweden | 29.7 | non-island | unitary | Yes | Limited contact tracing | limited testing |
| Switzerland | 55.9 | non-island | federation | Yes | Limited contact tracing | limited testing |
| Syria | 29.5 | non-island | unitary | No | No contact tracing | limited testing |
| Taiwan | 89.9 | island-like | unitary | No | Extensive contact tracing | symptomatic testing |
| Tajikistan | 56.6 | non-island | unitary | No | Limited contact tracing | limited testing |
| Tanzania | 35.2 | non-island | unitary | No | Limited contact tracing | No testing policy |
| Thailand | 84.5 | non-island | unitary | No | Extensive contact tracing | symptomatic testing |
| Trinidad and Tobago | 53.8 | island-like | unitary | No | Extensive contact tracing | limited testing |
| Tunisia | 61.9 | non-island | unitary | No | Extensive contact tracing | limited testing |
| Turkey | 58.0 | non-island | unitary | Yes | Extensive contact tracing | symptomatic testing |
| Uganda | 76.3 | non-island | unitary | No | Limited contact tracing | limited testing |
| Ukraine | 49.3 | non-island | unitary | No | Extensive contact tracing | limited testing |
| United Arab Emirates | 75.6 | non-island | federation | No | Extensive contact tracing | public testing |
| United Kingdom | 31.5 | non-island | unitary | Yes | No contact tracing | limited testing |
| United States | 40.8 | non-island | federation | Yes | Limited contact tracing | public testing |
| Uruguay | 72.1 | non-island | unitary | No | Limited contact tracing | limited testing |
| Uzbekistan | 82.4 | non-island | unitary | No | Extensive contact tracing | limited testing |
| Venezuela | 69.7 | non-island | federation | No | No contact tracing | public testing |
| Vietnam | 90.2 | non-island | unitary | No | Extensive contact tracing | public testing |
| Yemen | 7.7 | non-island | unitary | No | No contact tracing | No testing policy |
| Zambia | 81.6 | non-island | unitary | No | Extensive contact tracing | limited testing |
| Zimbabwe | 59.3 | non-island | unitary | No | Limited contact tracing | limited testing |
