# Supplementary material for: A novel comprehensive metric to assess effectiveness of COVID-19 testing: Inter-country comparison and association with geography, government, and policy response
Source: PLoS One. 2021 Mar 5;16(3):e0248176. doi: 10.1371/journal.pone.0248176 (PMC7935311; doi:10.1371/journal.pone.0248176)
Supplement: S1 Appendix — Eight alternative scenarios were considered. In each scenario, CovTI (and all subsequent inputs) was computed. Then, the MLR was run. MLR results were compared to the final model’s results. (DOCX) [file pone.0248176.s003.docx]

**S1 Appendix. Sensitivity analysis using alternative scenarios of model construction.**

Five choices in the model’s construction were evaluated using a sensitivity analysis. For each choice, at least one alternative model construction was evaluated (creating a new scenario). The alternative choice was chosen to be within the reasonable possible range for that parameter. In a sensitivity analysis, if variations of a model’s construction do not change within reasonable variations, then the model is considered robust, providing additional evidence for confidence in the results. The given choices considered in the sensitivity analysis were:

- a CFR that is capped to be no greater than 2x the ratio of D:C. (Eq 1);
- a 1% IFR, which factors into the 100D/C value in (Eq 5);
- the value of _mdemsys_, computed from I_dem_ and I_sys_ (Eq 6)
- 5% as the representative value for TPR at which testing is sufficient (Eq 7)
- the weighting factors for the CovTI sub-indices (Eq 13)

Based on those choices, the following scenarios were developed:

- If the CFR cap was 1.5x (A1) or 2.5x (A2) C/D, rather than 2x C/D (Eq 1)
- If the IFR was 0.5% (B1) or 0.75% (B2), rather than 1% (Eq 5)
- If the m_demsys_ was 10% greater (C1) or less (C2) than the value computed in (Eq 6)
- If the TPR threshold was 10% (D1) instead of 5%. (Eq 7)
- If equal weighting was given to each sub-index(E1) (Eq 13)

The mathematical implementation of each scenario is summarized in the table below.

| Scenario | Description |
| --- | --- |
| A1 | Change 2D/C to 1.5D/C in Eq 1 |
| A2 | Change 2D/C to 2.5 D/C in Eq 1 |
| B1 | Change 100D/C to 200D/C in Eq 5 |
| B2 | Change 100D/C to 133D/C in Eq 5 |
| C1 | Multiply mdemsys by 1.1 (Eq 6) |
| C2 | Multiply mdemsys by 0.9 (Eq 6) |
| D1 | Change TPR/0.05 to TPR/0.1 in Eq 7 |
| E1 | Change weighting of sub-indices to equal (25% each) (Eq 13) |

The robustness of the model’s conclusions were then assessed in two different ways by looking at how it would affect the MLR analysis. The adjusted R^2^ value and the F-statistic from the MLR were compared among the difference scenarios to see whether they changed substantially (i.e., by more than 10%).

| Scenario | F-statistic | Adjusted R^2^ |
| --- | --- | --- |
| Final | 7.07 | 0.225 |
| A1 | 7.04 | 0.224 |
| A2 | 7.11 | 0.227 |
| B1 | 7.59 | 0.240 |
| B2 | 7.31 | 0.232 |
| C1 | 7.11 | 0.227 |
| C2 | 7.01 | 0.224 |
| D1 | 7.40 | 0.235 |
| E1 | 6.86 | 0.219 |

As shown in the results, the results under all eight scenarios did not change the conclusions of the study (F-Statistic and Adjusted R^2^ within 10% of the final model’s values).

That is, CovTI still correlated strongly with the testing and contact tracing policies, as well as islands. This sensitivity analysis demonstrates that the model is robust and the factors driving the results are the raw data values and not the assumptions made in computing CovTI.
