# Supplementary material for: A novel comprehensive metric to assess effectiveness of COVID-19 testing: Inter-country comparison and association with geography, government, and policy response
Source: PLoS One. 2021 Mar 5;16(3):e0248176. doi: 10.1371/journal.pone.0248176 (PMC7935311; doi:10.1371/journal.pone.0248176)
Supplement: Attachment — Submitted filename: Review.pdf [file pone.0248176.s004.pdf]

Review to Manuscript: A novel comprehensive metric to assess COVID-19 testing outcomes: Effects of geography, government, and policy response

The manuscript proposes a comprehensive metric (CovTI) to evaluate the effectiveness of COVID-19 testing and allow comparison between different countries. The proposed index takes into account several indicators, including social and political indicators related to health system and level of democracy, and indicators related to the epidemics like the case fatality ratio, the proportion of active cases, the test positivity rate and the total number of tests. A multilinear regression model is proposed to relate this metrics with several different factors. It is shown that the proposed index highly correlates with the testing and contact tracing policy, and in lower measure with the geographical setting.

I appreciated the focus of the manuscript, which addresses a very interesting and important issue for evaluating the reactions to COVID-19 pandemic. I also appreciated the quality of the work. The validity of the proposed Testing Index is argued from a pragmatic point of view in the discussion. However, I would have appreciated more clarity and more solid justifications for the construction of the testing index, as well as some further comment on which results follow naturally from the construction and which ones indeed contain new information.

The authors stress as one of the main quality of the model is to be parsimonious. However, although each single element is clearly interpretable, I find that the construction of the overall metric lacks transparency, due to the many operations that connect each indicator with the final index. The risk is that the construction hides the dependence of the final metric on the different original indicators: some correlations are in fact inbuilt in the construction (e.g., lower Active Cases implies higher TI) while others may be intrinsic (e.g., the effect of the democracy index in the definition of the undetected epidemic), but their final effect is unclear.

Some more detailed comments follow.

- I think it would be useful to provide some justification to several choices in the mathematical construction of the Testing Index. For instance, while estimating the true number of infections, the reason for keeping separated the effects of I_sys, I_dem and D/C (that enter in the calculation of f1) from the test per capita and positivity rate (that enter in f2) is unclear. Also, it is unclear why taking the maximum rather than combining the indicators together in a sum or product.
- The reasons for taking the square root (e.g. in equations (5) and (6)) are not satisfactorily justified, as any increasing functions would play a similar role. Also the reason for (10) is not explained satisfactorily, and similarly many other choices throughout the manuscript. Further justification would also be appreciated for the relative weighting of the sub-indices playing a role in the final testing index.
- It is somehow unclear to me when the paper refers to cumulative quantities or to daily counts. For instance, it is unclear how to interpret the TPC (test per capita) indicator, and in particular the choice of using 100,000 as a separator (after (9)). When computing the TPR, I would think that the quantities are intended as cumulative (cumulative tests, cumulative cases). However,

using a threshold of 100,000 at any point in time, without considering the evolution of the epidemic, is not completely reasonable to me: countries where the epidemic started later would have less cumulative testing per capita than countries where the epidemic started earlier.

- P. 17, lines 4-5: this result is expected by how the CovTI was defined in the previous section. I think it would be useful to comment if and how it is expected or otherwise stress further why it is interesting.
- Only the derivation of the Detection Rate is corrected for the democracy index. However, transparency in data may also affect other aspects like the Case Fatality Rate or the number of Active Cases, both of which play an important contribution to the overall testing index. I wonder if authoritarian governments may have interest in hiding the death counts and active cases, hence resulting in an improved Testing Index (page 23, lines 20-21) because of lower CF (see also lines 24-26) and AC.
- The index depends on the current prevalence of the disease: the higher the prevalence, the lower the index. I am wondering if the conclusion about the index increasing from April to June (p. 19, lines 3-4) reflects a general downward trend of the epidemic in many countries, rather than a true indication of an improved testing
- There are two equations (12), one should be (13)
