# Supplementary material for: A novel comprehensive metric to assess effectiveness of COVID-19 testing: Inter-country comparison and association with geography, government, and policy response
Source: PLoS One. 2021 Mar 5;16(3):e0248176. doi: 10.1371/journal.pone.0248176 (PMC7935311; doi:10.1371/journal.pone.0248176)
Supplement: Attachment — Submitted filename: PONE-D-20-20527_R2R.docx [file pone.0248176.s005.docx]

**Response to Comments (RTC)**

**Ms. Ref. No.: PONE-D-20-20527**

Title: A novel comprehensive metric to assess COVID-19 testing outcomes: Effects of geography, government, and policy response

**Response to Editor’s Comments**

1.- Please ensure that your manuscript meets PLOS ONE's style requirements, including those for file naming.

We have done our best to conform to PLOS ONE’s style requirements. We have included the manuscript reference number in the file name in our revised submission.

2.- We note that the figures in your submission contain [map/satellite] images which may be copyrighted. All PLOS content is published under the Creative Commons Attribution License (CC BY 4.0), which means that the manuscript, images, and Supporting Information files will be freely available online, and any third party is permitted to access, download, copy, distribute, and use these materials in any way, even commercially, with proper attribution. For these reasons, we cannot publish previously copyrighted maps or satellite images created using proprietary data, such as Google software (Google Maps, Street View, and Earth). We require you to either (1) present written permission from the copyright holder to publish these figures specifically under the CC BY 4.0 license, or (2) remove the figures from your submission:

We have reviewed our images to ensure that we comply with copyright requirements. Currently, the “striking image” is the only map in our manuscript. However, we do not use any proprietary software or images in this map. The map was generated using open-source QGIS and open-source basemap files.

3.- We note that you have indicated that data from this study are available upon request. PLOS only allows data to be available upon request if there are legal or ethical restrictions on sharing data publicly.

In your revised cover letter, please address the following prompts:

a) If there are ethical or legal restrictions on sharing a de-identified data set, please explain them in detail (e.g., data contain potentially identifying or sensitive patient information) and who has imposed them (e.g., an ethics committee). Please also provide contact information for a data access committee, ethics committee, or other institutional body to which data requests may be sent.

b) If there are no restrictions, please upload the minimal anonymized data set necessary to replicate your study findings as either Supporting Information files or to a stable, public repository and provide us with the relevant URLs, DOIs, or accession numbers. Please see http://www.bmj.com/content/340/bmj.c181.long for guidelines on how to de-identify and prepare clinical data for publication. For a list of acceptable repositories, please see http://journals.plos.org/plosone/s/data-availability#loc-recommended-repositories.

Thank you. We have provided responses to these prompts in our revised submission’s cover letter. To reiterate those responses: a) There are no ethical or legal restrictions on sharing our data set. The raw data that we use is non-individualized publicly available data. Should you have any questions, you may contact the Khon Kaen University Center for Ethics in Human Research at +66(0)89-7141177 (<https://eckku.kku.ac.th/home>). b) We have opted to provide our data as part of the Supporting Information files. The full dataset has been uploaded as Supporting Information as comma-separated file (.csv).

**Response to Reviewer #1’s Comments**

Thank you for taking the time to review the manuscript. Your comments are very cogent and serve to greatly improve the integrity of our manuscript. Reviewer comments are in blue and responses are in black, with line numbers where appropriate

1. I appreciated the focus of the manuscript, which addresses a very interesting and important issue for evaluating the reactions to COVID-19 pandemic. I also appreciated the quality of the work. The validity of the proposed Testing Index is argued from a pragmatic point of view in the discussion. However, I would have appreciated more clarity and more solid justifications for the construction of the testing index, as well as some further comment on which results follow naturally from the construction and which ones indeed contain new information.

Thank you for your comments. We have increased the clarity and justification in the construction of the index improving our methodology in the following ways:

1. We changed the inclusion criteria. Now, only countries with >100 confirmed cases were included. The purpose is to exclude countries where essentially no outbreak has occurred (and, thus, assessing their response would not be appropriate). This reduced the sample size from 188 to 165 countries. Text describing this was added page 6, Lines 1-6.

2. We changed the way the DR is computed. It's simplified. Two simplifications: (a) The multiplier that adjusts for democracy and health system has been combined into a single multiplier that uses a linear relationship. The relationship was determined from regression to an existing model that estimated the true number of infections in around 35 countries. Text describing this was added around Page 9, Lines 4-10 (b) Tests per Capita (TPC) was completely removed. TPR is appropriate (proportional to outbreak) while TPC is not appropriate (proportional to population). The threshold for TPR, however, was reduced from 10% to 5%. The WHO had given this range of 5-10%, and in retrospect, most countries have aimed to be lower than 5%. Text describing these changes was added to Page 10, Lines 4-15.

3. We changed the way the index score is computed from the key indicators. In the revised form, the formulas that convert the indicator value into an index score are meant to mimic the percentile. Previous indices from other authors were constructed by making a score of 0 to 100 by percentile ranking (so the best country gets 100 and the worst gets 0). Following this method exactly, however, only provides relative information. One can probably imagine many scenarios where this would fail to appropriately assign scores (for example, every country is doing really well or really terribly). So, instead, we created formulas that follow the distribution of percentile rankings but still keep a score of 100 as the "ideal" value and 0 for the worst possible outcome. Text describing these changes was added to Page 11 Lines 11-21. Additionally, a new figure (Fig 2) illustrates these relationships.

4. We made a small change in the way the results were analyzed. Previously, the form of government was categorized into 3 groups, with one category being "authoritative government." That category was defined, however, based on the democracy index, which is inherent in the outcome variable (CovTI). This was not appropriate, so the analysis examines the influence of government through the binary lens of federation and unitary governments.

2. The authors stress as one of the main quality of the model is to be parsimonious. However, although each single element is clearly interpretable, I find that the construction of the overall metric lacks transparency, due to the many operations that connect each indicator with the final index. The risk is that the construction hides the dependence of the final metric on the different original indicators: some correlations are in fact inbuilt in the construction (e.g., lower Active Cases implies higher TI ) while others may be intrinsic (e.g., the effect of the democracy index in the definition of the undetected epidemic), but their final effect is unclear.

Thank you for this very important point. We have reworked several aspects of how the index is constructed in order to address your valid concerns. Overall, the goal was to remove unnecessary complications and to use more justifiable means to arrive at the final index. In addition, we added a figure (Fig 1) to show all operations and connections between them. These changes are noted in response to comments (RTC) #1.

With regard to the risk that the index’s construction obscures influence of certain input data, we don’t believe this is occurring. Probably the biggest concern would be that the proxy inputs, namely I_dem_ and I_sys_, would strongly influence CovTI, which would make CovTI a de facto measure of these indices, rather than a reflection of testing. To assess whether this was occurring, we regressed the index against these inputs. Scatterplots are shown below. What we see is that there is virtually no correlation between I_dem_ and I_sys_ and CovTI (adjusted R^2^ < 0.02 for both). On the other hand, the goal is for CovTI to be a reflection of epidemiological indicators that reflect testing effectiveness, namely DR, CFR, TPR, and AC. Therefore, it was our intention that the index be dependent on the epidemiological indicators.

3. I think it would be useful to provide some justification to several choices in the mathematical construction of the Testing Index. For instance, while estimating the true number of infections, the reason for keeping separated the effects of I_sys, I_dem and D/C (that enter in the calculation of f1) from the test per capita and positivity rate (that enter in f2) is unclear. Also, it is unclear why taking the maximum rather than combining the indicators together in a sum or product.

Thank you for your comment. Overall, we have added more justification and explanation for our choices. Generally, the aim to use the f1 factor (related to CFR, as adjusted by the democracy/health system factors). As noted in RTC#1, we have revised the methods to better justify our approach in computing f1. However, the alternative factor (f2) is used for unusual circumstances where a country had an extremely high TPR. This alternative factor f2 was used in 15 (9%) countries. If these two factors are viewed as means for assessing the same thing but through two different lenses, we believe it is logical to use the maximum of the two, rather than a multiplicative or cumulative effect. Changes in text at Page 10 Lines 4-11 were made to reflect these points.

4. The reasons for taking the square root (e.g. in equations (5) and (6)) are not satisfactorily justified, as any increasing functions would play a similar role. Also the reason for (10) is not explained satisfactorily, and similarly many other choices throughout the manuscript. Further justification would also be appreciated for the relative weighting of the sub-indices playing a role in the final testing index.

As detailed in RTC#1, we revised the approach to incorporating the effect of I_dem_ and I_sys_ by calibrating it to an existing model that estimated *Inf*. So, two separate equations using the square root function are no longer used. Instead, we now have equation 6 (m_demsys_), which is linear. Briefly, we used an existing model that used machine learning to estimate true number of infections in select countries as a way to calibrate the relationship between our democracy/health system indices and the missing number of infections (after accounting for the CFR, as used in our model). We found the relationship to be more linear and so we used the linear relationship now described. We have removed the TPC (previous equation 10), since it was an arbitrary and unnecessary complication. In fact, testing should be proportional to the outbreak and not the population.

In response to the weighting, we would refer to the approach used in a vulnerability index for the management of and response to COVID-19 in India. (<https://www.thelancet.com/journals/langlo/article/PIIS2214-109X(20)30300-4/fulltext>)

There, the authors (with precedent) used equal weighting in the absence of a rationale for weighting. We followed the same approach, except that we have categorized the underlying indicators in the index (i.e., the four domains) into two groups: 1) direct and 2) indirect. If CovTI is a measure of testing effectiveness and testing effectiveness is whether or not infections are detected as cases, the DR is a direct measure of that. On the other hand, CFR, TPR, and AC are indirectly related to testing effectiveness. Therefore, we gave equal weighting but double emphasis on the directly-related domain (i.e., DR). A statement was added on Page 11, Line 21. In addition, details were given in each sub-index (e.g., Page 12, Line 13).

5. It is somehow unclear to me when the paper refers to cumulative quantities or to daily counts. For instance, it is unclear how to interpret the TPC (test per capita) indicator, and in particular the choice of using 100,000 as a separator (after (9)). When computing the TPR, I would think that the quantities are intended as cumulative (cumulative tests, cumulative cases). However,

using a threshold of 100,000 at any point in time, without considering the evolution of the epidemic, is not completely reasonable to me: countries where the epidemic started later would have less cumulative testing per capita than countries where the epidemic started earlier.

This is a very good point. Originally, the purpose of the TPC was to provide some metric for countries where little to no outbreak had occurred early in the pandemic, and this remained in the index as a remnant. After careful consideration, we completely removed TPC as a metric used in computing the CovTI. In fact, testing needs to be proportional to the outbreak and not proportional to the population. To further avoid confounding effects of small/limited outbreak (which are not a fair comparison to countries with extensive outbreaks), we have limited the analysis to countries with more than 100 confirmed cases. This threshold was chosen as an arbitrary designation of countries considered to have sufficient outbreak for their testing to be evaluated.

6. P. 17, lines 4-5: this result is expected by how the CovTI was defined in the previous section. I think it would be useful to comment if and how it is expected or otherwise stress further why it is interesting.

Yes, this is a good point. Primarily, we want to illustrate that CovTI can holistically rank countries among several epidemiological indicators. We added some discussion to Page 22 Lines 2-7 and Page 24, Lines 5-8..

7. Only the derivation of the Detection Rate is corrected for the democracy index. However, transparency in data may also affect other aspects like the Case Fatality Rate or the number of Active Cases, both of which play an important contribution to the overall testing index. I wonder if authoritarian governments may have interest in hiding the death counts and active cases, hence resulting in an improved Testing Index (page 23, lines 20-21) because of lower CF (see also lines 24-26) and AC.

Thank you for this comment. We have decided to remove analysis that consider authoritarian governments. The main reason is that the index (dependent outcome) is inherently a function of the democracy index (which was used to define the independent variable—authoritarian governments). Therefore, this analysis would not be appropriate. Instead, we have analyzed only two types of governments—either unitary or federation—which are independent of any factor included in the calculation of the CovTI.

In general, you have a point that each type of input data (cases, tests, deaths), suffers from inconsistent definitions, reporting methods, and measurement techniques from country to country. Therefore, it is expected that some inter-country variability is due to this. We opted to include the adjustments for I_dem_ and I_sys_ in DR only. We could introduce excessive uncertainty or overparameterization into the model by adjusting all data in this way. Said another way, we will take the CFR, AC, and TPR for face value, since they are explicit epidemiological indicators implicitly reported by each country. On the other hand, DR must be estimated/modeled.

Hopefully by no longer analyzing the association with authoritarian governments and, thus, no longer needing to comment on the role of such government, we have sufficiently addressed this comment.

8. The index depends on the current prevalence of the disease: the higher the prevalence, the lower the index. I am wondering if the conclusion about the index increasing from April to June (p. 19, lines 3-4) reflects a general downward trend of the epidemic in many countries, rather than a true indication of an improved testing

This is a very good point. We have decided to remove the analysis and discussion about temporal changes in the index. Our primary goal was to develop and test the index and to identify factors associated with it. The temporal change, we considered, was not contributing enough to our goals to warrant inclusion in the manuscript.

9. There are two equations (12), one should be (13)

Thank you for catching this error. We have ensured that all equation numbers now count sequentially.
