# Supplementary material for: A novel comprehensive metric to assess effectiveness of COVID-19 testing: Inter-country comparison and association with geography, government, and policy response
Source: PLoS One. 2021 Mar 5;16(3):e0248176. doi: 10.1371/journal.pone.0248176 (PMC7935311; doi:10.1371/journal.pone.0248176)
Supplement: Attachment — Submitted filename: Response to Reviewers 020221.docx [file pone.0248176.s006.docx]

**Response to Comments**

**Ms. Ref. No.: PONE-D-20-20527R1**

Previous Title: A novel comprehensive metric to assess COVID-19 testing outcomes: Effects of geography, government, and policy response

Revised Title: A novel comprehensive metric to assess effectiveness of COVID-19 testing: Inter-country comparison and association with geography, government, and policy response

**Response to comments by Reviewer #3**

Because the comments did not give reference to specific lines/sections, we found it difficult to identify what methodological weaknesses, if any, were identified. This presented a challenge in determining how to proceed and how to adequately address the comments. Nevertheless, we used the opportunity to conduct our own thorough review, while considering every possible interpretation of the comments provided. We hope that our thorough response and subsequent edits will be sufficient to satisfy you.

1. Is the manuscript technically sound, and do the data support the conclusions? No.

We have attempted to defend the technical soundness of our approach by demonstrating the justification for our choices, the robustness of the model, and the multiple lines of evidence that support the validity of the model. We have attempted to address every comment on methodology below. We note that no comments were provided for the Discussion section or which seemed to critique the conclusions made from our findings.

2. Has the statistical analysis been performed appropriately and rigorously? No.

While no details were provided here, the comments below identified concerns with uncertainty or incorporation of possible stochasticity of the data. In addition to our responses below, we note that the multiple linear regression model and bivariate analyses report confidence intervals, which is standard practice. In addition, we believe we have followed appropriate statistical methods in our analysis. Furthermore, the sensitivity analysis (described below) demonstrates the robustness of the model.

3.This paper develops a metric to assess COVID-19 testing outcomes expressed as a linear combination of the rates CFsi, TPsi, ACsi and DRsi. These rates are computed with adjusted reported death data, the health system capacity and level of democracy of the countries. The idea of setting up a metric to compare the level of effectiveness of COVID tests in different countries is of major interest.

Thank you for recognizing the utility of a metric that quantitatively assesses the effectiveness of testing policies and detection of infections. We note that policy institutes continue to try to develop similar metrics, even as recently as January 27 with the release of the Lowy Institute’s Covid Performance Index. Other metrics include the Risk of Openness Index from Oxford University (links below). While no metric has directly captured the outcomes, we are trying to capture, we believe these efforts demonstrate the desire of policy makers and scientists to assess these factors and the demand of researchers to have a useful metric.

<https://interactives.lowyinstitute.org/features/covid-performance/>

<https://www.bsg.ox.ac.uk/research/publications/risk-openness-index-when-do-government-responses-need-be-increased-or>

We note the term “linear combination of rates” in reference to the sub-index values (CFsi, TPsi, ACsi, and DRsi). Considering the comments below about “linear relationships,” we wish to clarify that CovTI is the sum the sub-indices and does not represent a “linear relationship.” We believe the computation of each sub-index using a formula that mimics the percentile ranking of each country in the given category (see Fig. 2) is consistent with the established practice of using percentiles to compute index scores from 0 to 100 (see Page 11, Lines 6-21 or Citations #37, 38).

4. However, the scientific content of the paper lacks rigor and seems to me very far from the framework and the level of requirement of the journal PLOS. In fact, the authors take into account mostly stochastic factors (such as the political measures concerning the tests which only changed overnight depending on the availability of medical means, especially during the first phase of the covid) to ultimately propose a deterministic indicator without any analysis of the uncertainties linked to the stochasticity of the input data.

We agree that PLoS and other peer-reviewed journals have an obligation to uphold scientific principles that demonstrate rigor. However, following the first major revision, every decision and model construction was justified with a citation, reference to precedent, and/or demonstration of an empirical relationship. In our opinion, this fulfils the definition of scientific rigor.

We actually spent a substantial amount of time trying to debate and decode the meaning of “stochastic factors” in this comment.” Without going into too much detail about what we debated, including whether this was in reference to stochastic modeling vs. deterministic modeling or to extrinsic factors external to the model or to a belief that the raw input data was in some way stochastic or even that the comment really meant “uncertainty” as opposed to “stochasticity,” we would ask you to consider the effort we put into trying to address every possible interpretation of this comment in the “*Detailed Response to Concerns Raised by Reviewer #3*” below. To address this comment, we added a section to the Methods (“Sensitivity Analysis” Page 15, Line 4 to Page 16, Line 3) and Results (“Model Robustness” Page 22, Lines 2-9). We also added a new Supplementary Information S3 (“Sensitivity analysis using alternative scenarios of model construction.”)

5. Some relationships have been considered linear without any justification and sometimes wrongly, for example, why is the real number of infected people linearly proportional to the number of reported cases?

We have addressed your comment about linear relationships in a separate section in the detailed response below.

6. I agree to the great interest to have a good metric to assess COVID-19 testing outcomes however, the one proposed in this paper seems not relevant and not rigorous. For all these reasons and despite the answers given to the first phase of the review, I will not recommend the publication of this result in PLOS ONE journal.

Please see our previous comments above and in more detail below.

**Detailed Response to Concerns Raised by Reviewer #3**

**Stochasticity**

Reviewer’s comment: “…the authors take into account mostly stochastic factors (such as the political measures concerning the tests which only changed overnight depending on the availability of medical means, especially during the first phase of the covid) to ultimately propose a deterministic indicator without any analysis of the uncertainties linked to the stochasticity of the input data.”

The first concern raised is the use of “mostly stochastic factors” while using “a deterministic indicator without any analysis of the uncertainties linked to the stochasticity of the input data.” Because the reviewer references “the political measures concerning the tests which only changed overnight,” we are left guessing what factors to which the reviewer is referring. There are at least two broad ways to interpret this comment, and we are unsure which interpretation is closest to the intended meaning. The following interpretations are possible, as we see it:

- First possible interpretation: The input data are stochastic. The input data consist of cumulative cases, deaths, recovered cases, active cases, population, and tests completed, as well as two indices representing health system preparedness and degree of democracy (see white boxes in Figure 1). We do not believe it would be appropriate to interpret the raw input data as stochastic. This is the raw data.
- Second possible interpretation: The epidemiological indicators are stochastic (See green boxes in Figure 1). Four epidemiological indicators were computed, which fed into the four sub-indices of final index (CovTI). All four indicators have universally accepted definitions. In the case of active cases and test positivity rate, there is no justifiable way to modify the definition, as it can be directly computed from the raw data (so, we believe, no stochasticity). In the case of case fatality rate (CFR) and detection rate (DR), however, we recognize that there is a level of uncertainty in these values.

In short, we would agree that there is “uncertainty” in some of the model’s epidemiological indicators (namely, CFR and DR). Thus, following a thorough review of the model with the intent to identify possible introduction of uncertainty, we identified five possible points in the computation of CovTI where we made a choice or there is uncertainty in the true value of a parameter. This included the choices to use:

- a CFR that is capped to be no greater than 2x the ratio of D:C. (Lines 1-3, page 7; Eq 1);
- a 1% IFR, which factors into the 100D/C value in Equation 5 (Line 15-25, page 8)
- a linear relationship between Idem and Isys and Inf (as reflected in mdemsys; Eq 6)
- 5% for the representative value for TPR at which testing is sufficient (Eq 7)
- the weighting factors for the CovTI sub-indices (Eq 13)

First, we should point out that all five choices were provided justification and explanations for the logic of those decisions (Page 6 L17-26, Page 8 L15-23, Page 9 L4-10, Page 9 L23-24, Page 13 L21-23).

Nevertheless, we recognize that there is always some level of uncertainty in a decision, and variations in the accuracy of assumed relationships could alter the outcomes or results (thus, the concept of uncertainty, or some might say stochasticity). In our experience, a sensitivity analysis is a good way to assess how robust a model is relative to the level of uncertainty. We conducted a sensitivity analysis to see how the outcomes of the model and the conclusions based on the results would change (or not change) within a reasonable range of possible values for those parameters. Specifically, we considered:

- If the CFR cap was 1.5x or 2.5x C/D, rather than 2x C/D (Eq 1)
- If the IFR was 0.5% or 0.75%, rather than 1% (Eq 5)
- If the mdemsys was 10% greater or less than the value computed in (Eq 6)
- If the TPR threshold was 10% instead of 5%. (Eq 7)
- If equal weighting was given to each sub-index (Eq 13)

| Scenario | Description |
| --- | --- |
| A1 | Change 2D/C to 1.5D/C in Eq 1 |
| A2 | Change 2D/C to 2.5 D/C in Eq 1 |
| B1 | Change 100D/C to 200D/C in Eq 5 |
| B2 | Change 100D/C to 133D/C in Eq 5 |
| C1 | Multiply mdemsys by 1.1 (Eq 6) |
| C2 | Multiply mdemsys by 0.9 (Eq 6) |
| D1 | Change TPR/0.05 to TPR/0.1 in Eq 7 |
| E1 | Change weighting of sub-indices to equal (25% each) (Eq 13) |

The robustness of the model’s conclusions was then assessed in two different ways by looking at how it would affect the MLR analysis. The adjusted R^2^ value and the F-statistic from the MLR were compared among the difference scenarios to see whether they changed substantially (i.e., by more than 10%).

| Scenario | F-statistic | Adjusted R^2^ |
| --- | --- | --- |
| Final | 7.07 | 0.225 |
| A1 | 7.04 | 0.224 |
| A2 | 7.11 | 0.227 |
| B1 | 7.59 | 0.240 |
| B2 | 7.31 | 0.232 |
| C1 | 7.11 | 0.227 |
| C2 | 7.01 | 0.224 |
| D1 | 7.40 | 0.235 |
| E1 | 6.86 | 0.219 |

As shown in the results, the results under all eight scenarios did not change the conclusions of the study. In other words, CovTI still correlated strongly with the testing and contact tracing policies, as well as islands. This sensitivity analysis demonstrates that the model is robust and the factors driving the results are the raw data values and not the assumptions made in computing CovTI.

We added text to the manuscript describing this sensitivity analysis (Page 15 L3- Page 16 L10).

We also added the details of this analysis to a Supplementary Information file.

**Linear Relationships**

The second major concern identified was that “Some relationships have been considered linear without any justification and sometimes wrongly, for example, why is the real number of infected people linearly proportional to the number of reported cases?”

Similar to the first comment, we were uncertain about the intended meaning and had to consider each possible interpretation. For information, linear models are only of relevance for Equation 4, 6 and 12.

- First possible interpretation: A reference to Equation 4. Here, the interpretation of Equation 4 should not be that *Inf* (the true number of infections) has a linear relationship to *C*. Instead, as stated in Lines 2-5 of Page 8, the correct interpretation is that in all countries, the number of cases *C* (being those that show disease symptoms) is some sub-set of *Inf*. Expressed another way, *C = (1/f)Inf*. That is, *C* is always some proportion of *Inf*, in which case (*1/f*) is a proportion from 0 to 1 (representing that the DR is somewhere between 0 and 100%). In this case, the value of *f* does not always follow a linear relationship. It can be computed using one of the two methods (f1 or f2). In this case, the comment that a linear relationship has not been justified is not an appropriate or relevant comment, since Equation 4 does not imply a linear relationship but rather an acknowledgement that C must logically be some proportion of *Inf* (C cannot be more than Inf, unless cases are being double-counted).
- Second possible interpretation: A reference to Equation 6. As stated in Lines 4 through 12 on Page 9, this relationship was determined by looking at another model’s estimates [See Citation #35] of the true number of infections in 35 countries (the dependent variable) and the two index values (Idem and Isys, the independent variables). Three possible relationships were considered and the linear model was the best fitting, resulting in Equation 6. In this case, we believe we have justified the use of a linear relationship.
- Aside from these possible interpretations, no other linear relationships were used. As stated in Lines 10-21 of Page 11, the mathematical relationships used to determine the sub-index values (Equations 9-12) were fitted to represent the distribution of the percentile rankings of each sub-index. They are not linear, except for Equation 12, which represents the pattern of percentiles (see Figure 2).

In response, we believe we have justified the choices for relationships used (including linear ones), especially as a result of the excellent feedback provided by Reviewer #2. Thus, we disagree with this second concern. Namely, few linear relationships are used. And, those that are used, we believe have been sufficiently justified. However, due to our uncertainty in the intended meaning, we cannot be sure if we are understanding the concerns correctly.

**Overall Response**

In summary, we believe the manuscript presents several lines of evidence that this parsimonious model captures the significant components of testing it intends to represent without overparameterization. First, this model’s estimates of DR are in line with comparable national estimates of period prevalence from epidemiological models and serological monitoring (see Table 2). Second, the countries that received high scores in CovTI, such of New Zealand, Vietnam, Taiwan, South Korea, Norway, and Thailand, are widely recognized as having effective testing programs combined with other effective public health responses (see Table 3). Thirdly, the strong association of testing and contact tracing policies with CovTI give empirical evidence that the metric is capturing the effects of testing and not confounding factors (see Tables 4 and 5).

In conclusion, we kindly ask you to consider whether we have satisfied the comments and demonstrated the scientific rigor of the manuscript and alleviated concerns over uncertainty/stochasticity.

**Unsolicited Revisions**

1. We decided to slightly revise the title to specifically identify that our metric facilitates inter-country comparisons (Page 1, Lines 1-2)

2. We identified an error in the caption of Table 5 (the value of the F-statistic was not updated from Submission 1 to Submission 2. (Page 21, Line 6)

3. We updated references. Some citations referenced pre-prints that have subsequently completed peer review and are published. In those cases, references were updated to cite the appropriate journal.
